# From iPSC to manufactured iNK cells using CombiCult® screening platform

**DOI:** 10.3389/fcell.2026.1824021

**Published:** 2026-06-11

**Authors:** Marina Tarunina, Sachin Luharia, Matthew Houppermans, Giuseppe D’Agostino, Lam Lam, Michelle Gestwa, Aleksandra Habich-Crayton, Marcia Mata, Juline Guenat, Molly Tregidgo, Patrick Statham, Charlotte Lee-Reeves, Mudith Jayawardena, Simona Zingaro, Limor Zwi-Dantsis, Aishwarya Nair, Alexandru-Robert Podovei, Vera Karels, Jahid Hasan, Tatyana Ponomaryov, Yen Choo

**Affiliations:** 1 Plasticell Ltd., Stevenage Bioscience Catalyst, Stevenage, United Kingdom; 2 Cell and Gene Therapy Catapult, Guy’s Hospital, London, United Kingdom; 3 Cancer Discovery and Regenerative Medicine, Lee Kong Chian School of Medicine, Singapore, Singapore

**Keywords:** human iPSc, immuno-oncology, manufacturing, STR, natural killer cells, off-the-shelf therapy

## Abstract

**Background:**

Allogeneic cell-based immunotherapies generated from pluripotent stem cells show considerable promise for the treatment of oncological, autoimmune, and viral diseases, however discovery platforms for induced pluripotent stem cell (iPSC)-derived cell therapies do not translate well to scalable manufacturing platforms.

**Methods:**

We applied a high-throughput combinatorial screening platform (CombiCult®) to identify novel, manufacturing-ready, feeder-free protocols for the generation of mature, functional NK cells from human iPSCs.

**Results:**

We validated seven CombiCult®-derived differentiation protocols for the production of highly cytotoxic, phenotypically mature iPSC-derived NK (iNK) cells, which are comparable to donor-derived NK cells. Translation to a Stirred Tank Bioreactor (STR) system resulted in a 10x increase in productivity, from ∼20 to ∼190 iNK cells per starting iPSC. iNK cells demonstrate mature transcriptomic signatures, retained after translation to bioreactor-based production.

**Conclusion:**

The three-dimensional, bead-based screening approach enables seamless translation to bioreactor-based production of iNK cells exhibiting high cytotoxic activity against a range of cancer cell types.

## 1 Introduction

The field of adoptive immune cell transfer has been undergoing a revolutionary transformation since the initial success and clinical implementation of autologous CAR-T cells for the treatment of hematologic malignancies. Innovative gene-engineering strategies are driving breakthroughs in advanced cellular immunotherapies by arming effector immune cells with molecular tools that target diseased cells with remarkable precision.

As of April 2026, the FDA had approved seven CAR-T cell therapies for various relapsed or refractory blood cancers, including selected lymphomas, leukemias, and multiple myeloma (FDA, 2026), and more than 1,500 CAR-T cell therapy clinical trials were registered worldwide (Cao et al., 2025). However, the challenges and limitations of these therapies have prompted the search for new approaches. Autologous CAR-T cells carry risks such as cytokine release syndrome and immune effector cell-associated neurotoxicity syndrome, whereas allogeneic CAR-T therapies face the potential for graft-versus-host disease (Cao et al., 2025). In contrast to T cells, allogeneic NK cells can be administered safely and effectively without strict human leukocyte antigen matching. Donor cell sources and manufacturing strategies vary, ranging from the NK-92 lymphoma cell line, through expansion of donor peripheral blood (PB)- or umbilical cord blood (UCB)-derived NK cells, to differentiation of UCB-derived CD34-positive progenitor cells (Romanini et al., 2025; Shah et al., 2026). Nevertheless, lengthy manufacturing times, batch-to-batch variability, and occasionally poor quality or insufficient quantities of primary donor cells impose significant constraints on the cost and reproducibility of cellular therapy products.

Human iPSCs represent an alternative, virtually unlimited source of allogeneic iNK cells, offering strong potential for generating large, reproducible stocks of “off-the-shelf” therapeutic products. Because iPSCs are highly amenable to genetic manipulation, genome-editing strategies can be implemented to enhance cytotoxicity while ensuring sufficient expansion and persistence of the iNK cell product (Boyd-Gibbins et al., 2022). Unlike T cells, which can give rise to long-lived memory populations, NK cells have only a limited capacity for prolonged survival and sustained functional activity in circulation, often necessitating repeated dosing with large cumulative quantities of effector cells (ranging from 10^8^ to 10^9^ NK cells per patient) (Bakhtiyaridovvombaygi et al., 2025).

Safety, reproducibility, efficacy, compliance with Good Manufacturing Practice guidelines, as well as yield and cost, are critical parameters assessed during the development of manufacturing processes for NK cell therapy products. Several iNK therapy candidates are currently in early-phase clinical trials and have demonstrated favourable safety profiles (Page et al., 2024). However, treatment success often depends on multiple cell infusions to maintain a sustainable number of effector iNK cells in circulation.

We have previously demonstrated the strategic advantage of CombiCult®, a bead-based screening technology, in enabling the design and discovery of highly efficient differentiation protocols for hematopoietic cell populations using iPSC clusters as a starting material (Tarunina et al., 2014; Tarunina et al., 2019; Tarunina et al., 2026). Here, we aimed to apply this platform to address several challenges in iNK production, including scalable manufacturing through three-dimensional (3D), feeder-free culture systems that generate a well-characterized pool of functional iNK cells targeting solid tumours.

## 2 Materials and methods

### 2.1 Reagents for media preparation

All reagents used in media compositions for CombiCult®, and validation experiments are summarized in the Supplementary Table S1.

### 2.2 Culture of hiPSC lines

Gibco human episomal iPSC line (Gibco, A18945) was used in this study.

iPSCs were cultured on culture vessels coated with vitronectin (rhVTN) (Gibco, A14700) in Essential 8 (E8) medium (Gibco, A1517001) at 37 °C in 5% CO2. iPSCs were passaged using Accutase (StemCell Technologies, 07920) and seeded in E8 medium supplemented with 10 μM Rho-kinase inhibitor Y-27632 (ROCKi) (Tocris Biosciences, 1254). ROCKi was removed from culture medium 24 h after seeding. Media were changed every day.

For expansion in Stirred Tank Bioreactors (STRs), iPSCs harvested from expanded cells on rhVTN-coated flasks were adapted to suspension culture in a BioBLU 0.3sc Single-Use Bioreactor (0.3L STR) (Eppendorf, 138610200) for a single expansion cycle before differentiation. On Day 0, cells were resuspended in 130 mL of E8 medium with 10 µM ROCKi and cultured at a constant rotational speed of 170 rotations per minute (rpm). After 24 h, clustered iPSCs were then supplemented with an additional 60 mL of E8 medium without ROCKi and perfusion was initiated using a cell retention device (BioSep acoustic filter, Getinge). On Day 5, clusters were ready for differentiation and were transferred into Step 1 medium. Clusters were sampled daily and transferred to a 6-well plate for imaging by phase contrast microscopy using the ×4 objective. Images were analysed using a machine learning algorithm called VAIDR (Thinking Research Instruments GmbH) according to a proprietary protocol and results transferred to GraphPad Prism for presentation.

### 2.3 CombiCult® screen

**FIGURE 1:**
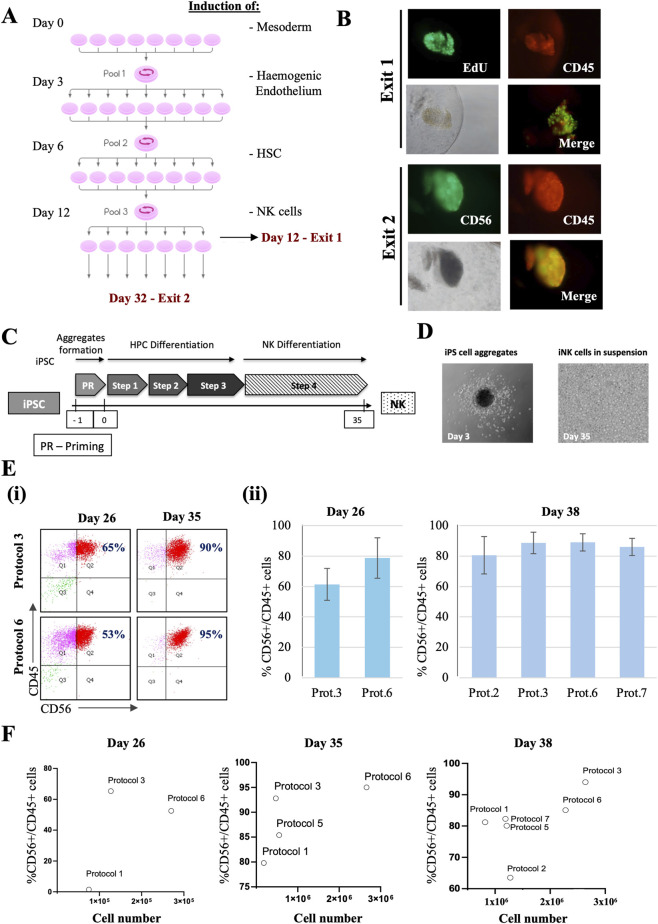
Development and selection of iNK differentiation protocols using CombiCult® screening platform. **(A)** CombiCult® matrix scheme showing stages of differentiation, split-pool schedule and number of conditions sampled. Beads were collected and processed at 2 Exits at Day 12 and Day 32. **(B)** Examples of positive beads sorted by bright fluorescent signal of Click-iT EdU and CD45 (Exit 1) and CD56/CD45 markers (Exit 2). **(C)** The timeline for differentiating iPSC into iNK cells used for validation studies. **(D)** Representative images of aggregate-based differentiation in static culture conditions. **(E)** Specification of iNK cells differentiated from iPSC using CombiCult®-derived protocols as shown by flow cytometry (i) and quantified for 5 independent experiments (ii). **(F)** Cell numbers of differentiated iNK cells per well (yield) were plotted against cell purity (%CD56+/CD45+ cells) (representative charts are shown for one of the experiments). Protocols with the highest yield and purity are considered the best production protocols.

A detailed description of the CombiCult® experiment can be found in the Supplementary Data. Briefly, on Day 0, mechanically harvested iPSC clumps were resuspended in a 2.0% (w/v) solution of sodium alginate (Sigma-Aldrich, 71238) in PBS. iPSCs were encapsulated into alginate beads by electrospraying using microencapsulator (Nisco). Method for producing cells of hematopoietic origin in alginate beads was previously developed at Plasticell and adapted for use in CombiCult® screening experiments (Tarunina et al., 2014; Tarunina et al., 2016; Tarunina et al., 2019). The beads were divided among 8 media conditions for Step 1 (Figure 1A; Supplementary Table S1). Approximately 7,500 beads (∼300 cells per bead) were tested in each Step 1 media formulation. On Day 3, beads were tagged, pooled together and distributed in media conditions for Step 2 (Figure 1A; Supplementary Table S1). This procedure was repeated on Day 6 for Step 3 (Figure 1A; Supplementary Table S1). On Day 10, part of the beads was incubated with EdU. On Day 12, beads incubated with EdU were collected and fixed for Exit 1 (Figure 1A). Remaining of the beads were tagged, pooled and distributed in media conditions for Step 4 (Figure 1A; Supplementary Table S1). On Day 32, all beads were fixed for Exit 2. Fixed beads were immunostained for proliferative hematopoietic cell markers CD45 and EdU (Exit 1) or NK cell phenotypic markers CD56+/CD45+ (Exit 2). Stained beads were sorted on COPAS (Union Biometrica). Tags from sorted positive beads were released and analysed by flow cytometer. Tag information carrying individual bead media history was deconvoluted and analysed by Plasticell’s proprietary software Ariadne^TM^ (Suppl. Methods).

### 2.4 Validation of CombiCult®-derived protocols in aggregate cultures

For differentiation in static conditions, iPSCs colonies were cut into fragments using StemPro EZPassage Disposable Stem Cell Passing Tool (Gibco, 23181010) on Day -1 (Figure 1C) and seeded in E8 medium supplemented with 10 μM ROCKi in 6 well suspension plate (Greiner, 657185) for aggregate formation. On Day 0, aggregates were transferred to a 37 μm cell strainer (StemCell Technologies, 27250). Flow-through containing smaller aggregates and cell suspensions was discarded. Larger aggregates were collected, transferred to Step 1 medium and seeded at a density between 50–70 aggregate per well in a 12-well TC-treated plate (Corning, 3513) for differentiation. Complete media change was performed at the beginning of each step of differentiation. Half media change was performed on Day 8, 10, and every 3–4 days from Day 12 onwards.

For differentiation in STR, iPSCs were seeded into 0.3L STR vessels and base medium supplemented with step-specific growth factors was changed according to Protocol 7 until Day 35. Clusters were differentiated at 37 °C in 5% CO2 and 170rpm agitation speed. The differentiation was carried out in a four-stage protocol. Complete media formulations and differentiation schedule for suspension differentiation are summarized in the Supplementary Table S2. For routine quality control (QC), aggregates after each stage were dispersed into single cells using pre-warmed Accutase and stored in MACStaining buffer (Miltenyi Biotec, 130-138-335) at 4 °C.

### 2.5 Flow cytometry analysis of differentiating cells

Cells were collected, filtered through a 70 μm strainer and stained with appropriate antibodies or isotype controls (Suppl. Methods). A BD FACSCanto^TM^ II flow cytometer (BD Biosciences) was used for acquisition. BD FACSDiva™ Software (BD Biosciences) and FlowJo v10 software (BD Biosciences) were used for the analysis of flow data.

### 2.6 Testing cytotoxicity of produced iNK cells *in vitro*

#### 2.6.1 Cell lines

NK-92 MI (ATCC, CRL-2408) cells were maintained in MyeloCult H5100 (StemCell Technologies, 05150) supplemented with 1 μM hydrocortisone and used as a positive control. T47D (ATCC, HTB-133) breast cancer cells expressing nuclear GFP reporter were maintained in RPMI-1640 Medium (Gibco, 11875093) with 10% Fetal Bovine Serum (FBS) (Gibco, 10500064). PANC-1 (ATCC, CRL-1469) pancreatic cancer cells expressing nuclear NIR FP reporter were maintained in DMEM (Gibco, 31966047) with 10% FBS. A549 (ATCC, CCL-185) lung cancer cells expressing luciferase reporter were maintained in DMEM with 10% FBS. Raji (ATCC, CCL-86) cells were maintained in RPMI-1640 Medium with 10% FBS. K-562 (K562) (ATCC, CCL-243) cells were maintained in RPMI-1640 Medium with 10% FBS.

#### 2.6.2 Translocation of CD107A upon activation

iNK cells or NK-92 cells were co-cultured with Raji cells at 1:1 ratio for 2 h in the presence of αCD107A Ab (BD Biosciences, 555800) and stained for CD56 (BD Biosciences, 562751). To set a positive control for the assay, NK cells were stimulated with Phorbol 12-myristate 13-acetate (PMA) (Sigma-Aldrich, P8139) and Ionomycin (Sigma-Aldrich, I0634) for 2 h in the presence of αCD107 Ab. Stained cells were acquired by FACSCanto^TM^ II flow cytometer.

#### 2.6.3 Determining cytotoxicity against adherent cancer cells

If not specifically indicated, the iNK cells produced by different protocols were collected at Day 40 of differentiation. Target cancer cells were seeded to 96 well plates, at 10,000 cells/well for T47D and PANC-1, and 15,000 cells/well for A549, 1 day prior to the addition of effector cells (NK cells) for cytotoxicity assay. Effector cells were added to target cells at various effector to target (E:T) ratios, ranging from 0.25:1 to 2:1. After 24 h and/or 48 h of co-culture of effector and target cells, relative percentage of live target cells remained in each well was measured and quantified.

#### 2.6.4 Data acquisition

For imaging-based assay, the 96 well plates were scanned using CellInsight CX5 high-content screening platform (ThermoFisher). Number of live target cells in each well was quantified based on the count of cells positive for the nuclear fluorescent protein. The number of live target cells in each condition was normalized to the number of live target cells in the condition without effector cells (i.e., cancer cells only), and the percentages of live cells were used to plot cytotoxicity graphs.

For luciferase-based assay, ONE-Glo Luciferase Assay System (Promega, E6120) was used according to manufacturer’s protocol. Luminescence was measured using CLARIOstar (BMG LABTECH). Total luminescence is proportional to the number of live target cells in each well. The total luminescence in each condition was normalized to the total luminescence in the condition without effector cells (i.e., cancer cells only), and the percentages of live cells were used to plot cytotoxicity graphs.

NK-92 cells were used as an assay control.

### 2.7 Activation of iNK cells by K562 cells with multiparametric flow cytometry detection

iNK cells were produced by different protocols and collected at indicated days of differentiation. iNK cells were co-incubated with K562 for 24 h in suspension at a ratio E:T = 1:2. UCB-derived NK cells were used as control for the assay. After co-incubation, the cells were collected, stained and acquired on Cytek Aurora flow cytometer for the multiparametric detection of changes in expression of activating and inhibitory molecules characteristic of NK cells cytotoxicity response. The expression of these molecules on unstimulated iNK cells was used to identify the baseline expression.

### 2.8 Determining expression profiles by single cell RNA sequencing (scRNAseq)

#### 2.8.1 scRNAseq sample preparation

iPSCs were thawed and recovered for one passage before fixation. Cryopreserved donor peripheral blood mononuclear cells (PBMCs), PB-CD34^+^ cells and UCB-CD34^+^ cells were thawed and recovered overnight before fixation. PBMC-NK were isolated from thawed and recovered donor PBMCs using NK cell Isolation Kit, human (Miltenyi Biotec, 130-092-657), according to manufacturer’s protocol. iNKs were collected from the differentiation experiment for fixation. All cells were fixed using Evercode^TM^ Cell Fixation v2 (Parse Biosciences, ECF2101), according to manufacturer’s protocol V2.1.2. Libraries for scRNAseq were prepared from fixed cells using Evercode^TM^ WT v2 (Parse Biosciences, ECW02135), according to manufacturer’s protocol V2.2.2. Number of fixed cells used for sample preparation was calculated using Parse Biosciences Evercode^TM^ WT Sample Loading Table v2 (Parse Biosciences), with targeted 2750 barcoded cells for each control sample (i.e., iPSC, PB-CD34^+^, UCB-CD34^+^, PBMC and PBMC-NK samples) and targeted 4125 barcoded cells for each iNK sample. Libraries were sequenced using service from Novogene (United Kingdom).

#### 2.8.2 scRNAseq data analysis

FASTQ files from 8 sublibraries were aligned and quantified using the Parse Biosciences split-pipe pipeline v. 1.2.0 with default arguments, using the Ensembl Grch38 release 108 reference genome annotation. This processing step resulted in 44,978 cells passing QC. The resulting count matrices were read in R v. 4.3.2 into a Seurat v 5.2.1 (Hao et al., 2024) object and cells were annotated according to their barcode 1, indicating the sample of origin (CD34^+^, NK, PBMCs, iPSCs, and all the other differentiation runs, each in its own replicate). Cells were discarded according to per-sample thresholds that were empirically determined as median +/− 3.5 median absolute deviations (MADs), using log10 (total UMIs per barcode), log10 (total genes detected), % mitochondrial transcripts (only above), and % MALAT1 transcripts (only above). This resulted in a total of 41,491 cells left for further analyses. Counts were normalized using sctransform v. 0.4.1(Hafemeister and Satija, 2019) with default parameters, and the transformed counts were used as input to Seurat’s RunPCA for dimensionality reduction. The 10 first principal components were retained for downstream reduction with RunUMAP, setting min.dist = 0.7. Cells without manufacturing runs were subset from the dataset and embedded in a different UMAP for visualization. Given the absence of batch effects due to the combinatorial barcoding of transcripts, no integration was carried out, and differential expression was tested between samples of different origin without re-clustering, using the fast Wilcoxon rank sum test with presto v. 1.0.0. In particular, the following comparisons were tested: Protocol 3 and Protocol 7 vs. PBMC NK, and Protocol 3 and Protocol 7 in static conditions vs all manufacturing conditions individually and in a pool. Genes were scored by logFC * −log10(padj) of the Wilcoxon rank sum test. For pseudotemporal trajectory inference monocle3 v. 1.3.4 (Cao et al., 2019) was used on the UMAP embedding of the STR and static timepoints, using default parameters and selecting as a starting point a cell in the day 15 sample. Differential expression across the trajectory was estimated via the testPseudotime function from TSCAN v. 1.40.1 (Ji and Ji, 2016), fitting changes along the pseudotime vector on a spline basis matrix with 5 degrees of freedom. All plots were generated using ggplot2 (Wickham, 2016), with the exception of pseudotime heatmaps which use ComplexHeatmap v. 2.18.0 (Gu et al., 2016).

### 2.9 Statistical analysis

Data are presented as a mean value for at least 2 independent samples ± standard deviation, analysed in Excel.

## 3 Results

### 3.1 CombiCult® screening platform is used for the discovery of feeder-free protocols for iNK production

In this study, we sought to identify robust, manufacturing-ready differentiation protocols for the generation of mature and functional induced natural killer (iNK) cells from human induced pluripotent stem cells (iPSCs). We have previously demonstrated that clumps or aggregates of iPSCs encapsulated in alginate beads give rise to hematopoietic lineage cells under feeder-free, three-dimensional (3D) culture conditions that mimic bioreactor environments (Tarunina et al., 2019; Tarunina et al., 2026).

Assay development for the CombiCult® screening experiment was performed using iPSC fragments encapsulated in alginate beads. Two distinct screening “Exits” were defined to enable selection at different stages of differentiation: Exit 1, targeting proliferating hematopoietic precursor cells at Day 12 of differentiation, and Exit 2, targeting mature NK cells at Day 32 of differentiation.

To establish the immunochemical detection assays, specific marker combinations were evaluated at each exit point. For Exit 1, the common hematopoietic marker CD45 was combined with a DNA-linked proliferation marker using the Click-iT EdU assay. For Exit 2, mature NK cells were identified based on expression of CD56, the major NK cell surface antigen, in combination with CD45 (Figures 1A,B).

Figure 1A presents a schematic overview of the CombiCult® Matrix design. Each step in the matrix comprises multiple defined media compositions that guide stepwise differentiation from iPSCs to iNK cells (Supplementary Table S1), progressing through mesenchymal, hematopoietic, and NK lineage specification during defined culture intervals according to the experimental timeline (Figures 1A,C). Combinations of WNT signalling activators, TGF-β and PI3K inhibitors, as well as VEGF, FGF and Activin ligands, were incorporated at the early stages of the Matrix to design optimal media compositions that promote mesoderm induction and hematopoietic priming conducive to NK cell differentiation. To enhance reproducibility and ensure compatibility with clinical manufacturing standards, we evaluated serum-free alternatives, including polyvinyl alcohol, recombinant human serum albumin, fatty acids, lipids, vitamins and SyntheChol. To eliminate the need for feeder cells such as OP9-DL1, which are typically used to activate Notch signalling during lymphoid priming and differentiation, we integrated various Notch ligands (DLL4 and Jagged1) directly into the Matrix. These ligands were often presented as Fc chimeras, either alone or in combination with IgG, to promote formation of higher-order multimers and enable modulation of Notch receptor signalling (Narui and Salaita, 2013; Biktasova et al., 2015). A human IL-15Rα fragment, a soluble sushi domain of IL-15 Receptor α, was included in several media formulations to improve the survival and proliferation of iNK cells through persistent stimulation of IL-15 signalling (Mortier et al., 2006).

In total, 32 distinct media compositions were applied sequentially to generate 4032 (8 × 9 × 8 × 7 = 4,032) differentiation protocols, which were evaluated for their ability to produce proliferating hematopoietic precursor cells at Exit 1 and mature iNK cells at Exit 2 (Figures 1A,B). Approximately 60,000 alginate beads (corresponding to 15 beads per protocol) were included in the screen. Following the split-pool workflow, beads were tagged and distributed across media compositions defined by each step in the CombiCult® matrix (Supplementary Table S1). For Exit 1 (Day 12), around 8,500 beads were incubated with Click-iT EdU, then fixed, stained, and analyzed by COPAS sorting to enrich for beads containing actively proliferating CD45-positive cells (Figure 1B; Supplementary Figure S1i). At the final screen endpoint (Exit 2, Day 32), remaining beads were fixed, stained, and sorted based on strong fluorescence corresponding to CD56/CD45-double positive cells (Supplementary Figure S1ii). In total, 102 and 109 positive beads were individually sorted into wells at Exit 1 and Exit 2, respectively (Figure 1B). Accumulated fluorescent tags were subsequently released from the sorted beads and identified by flow cytometry. Ariadne™ bioinformatics analysis revealed that approximately 65% of the analyzed beads exhibited complete tagging and demonstrated strong clustering among protocols, with some clusters overlapping between Exit 1 and Exit 2, representing good candidates for best-hit selection (Supplementary Figure S1iii). Seven candidate differentiation protocols were selected for further validation (Supplementary Table S2).

### 3.2 Validation of CombiCult®-derived protocols for iNK production in aggregate cultures

Aggregate cultures were initiated from iPSCs in complete iPSC culture medium supplemented with ROCKi (Priming phase; Figure 1C). Defined media formulations were applied sequentially at each differentiation stage in accordance with the protocols selected for validation (Supplementary Table S2). Complete medium exchanges were performed on Days 0, 3, 6, and 12 following the experimental timeline (Figure 1C).

Cell cultures were routinely monitored by light microscopy. The emergence of suspension cells at approximately Day 6 was indicative of hematopoietic differentiation (Figure 1D). From Day 12 onward, suspension cells were harvested and analysed for hematopoietic stem and progenitor cell (HSC/HSPC) markers. By Day 12, up to 30% of cells expressed CD34. While differentiation progressed, a steady increase in CD43 and CD45 expression was observed, concomitant with a decline in CD34 expression (Supplementary Figure S2A).

Cells were subsequently maintained in Step 4 maturation medium to promote NK cell differentiation until Day 35 or beyond. Suspension cells (Figure 1D) were collected, counted, and analysed by flow cytometry for expression of CD56, CD45, and CD3 to assess the purity and yield of developing iNK cells (Figure 1E). All analysed populations were CD3-negative, excluding contamination by CD56-positive/CD3-positive NKT cells for at least up to 60 days in culture (Supplementary Figure S2B).

To facilitate selection of optimal differentiation conditions, cell yield was plotted against cell purity (percentage of CD56/CD45-double positive cells) across the maturation period for each validated protocol. The results indicate that the validated protocols varied in their capacity to generate populations of mature iNK cells. Protocol 3 (4-4-7-1) and Protocol 6 (4-7-2-1), which shared Step 1 and Step 4 differentiation media, produced the best yield and purity across all tested differentiation timepoints (Days 26, 35 and 38). In both protocols, the differentiating iNK cells were exposed to Notch signalling activator DLL4 and Human IL-15Rα sushi protein from Day 12 to Day 38 of differentiation. Among the other protocols, serum-free Protocol 5 (3-1-5-3) and serum-dependent Protocol 7 (6-7-8-6) delivered good iNK cell purity at Day 38 of differentiation but lagged in iNK yield. This analysis enabled identification of the best-performing differentiation protocols for iNK cell production in aggregate culture systems (Figure 1F).

### 3.3 Aggregate iPSC cultures produce mature iNK cells

The cytotoxic activity of NK cells is governed by the balanced expression of surface receptors involved in target recognition and cellular activation. The coordinated expression of activating, inhibitory, adhesion, cytokine, and chemokine receptors, together with a fully developed intracellular effector machinery capable of responding to activating stimuli, define both the maturation state and functional capacity of iNK cell products intended for cell therapy applications.

**FIGURE 2:**
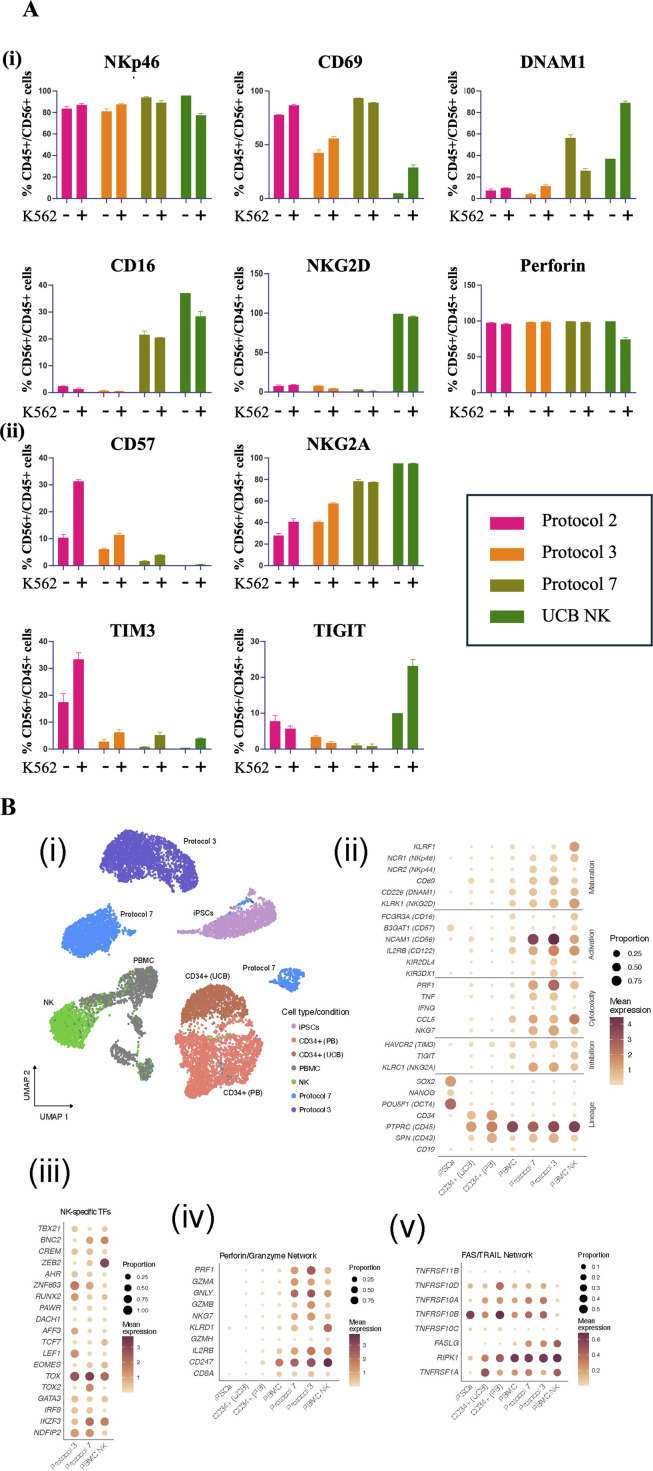
iNK cells produced in static 2D conditions resemble donor-derived NK cells. **(A)** Phenotypic analysis of iNK cells showing the expression of activating (i) and maturation/inhibitory (ii) receptors as analyzed by FACS. The intracellular staining for Perforin was performed on fixed and permeabilized cells. iNK cells were collected at Day 49 (Protocols 2 and 3) and Day 42 (Protocol 7) of differentiation. All measurements were compared to UCB-NK. NK cells were exposed to K562 for 24 h (“+”) or left untreated (“−”). **(B)** Gene expression profile derived from scRNAseq analysis of iNK cells produced in static cultures. (i) UMAP embedding of the single cell transcriptomes for static conditions, PBMCs, PBMC-derived NKs, iPSCs, and CD34^+^ from UCB and PB. Each point represents a single cell, coloured by the sample of origin. The UMAP coordinates show that each sample/tissue type clusters separately. (ii) Dot plot showing the mean log-normalized expression and percentage of expressing cells of genes relevant for different aspects of NK differentiation and function, including (iii) transcription factors (TFs), (iv) perforin/granzyme network genes, and (v) FAS/TRAIL network genes.

Multiparametric flow cytometry analysis revealed high surface expression of key activating receptors, including NKp46, NKp44, DNAM-1, and CD69, on iNK cells (Figure 2A). Perforin, the effector molecule involved in killing of target cells, was expressed at high levels in almost 100% of iNK cells produced by all studied protocols, as revealed by intracellular immunofluorescence. Moderate to low expression levels of NKG2A, CD57, TIGIT, and Tim3 suggested reduced inhibitory activity of these receptors on the cytotoxic potential of iNK cells (Figure 2A). Clear trends were observed in surface marker expression among cell populations generated by different protocols. The expression of CD16, DNAM and NKG2A was more prominent in iNK cells produced by Protocol 7, indicating a greater similarity with donor NK cells. NKp46 and perforin were highly expressed in both iNK and donor NK cells, while early activation marker CD69 was highly expressed only in iNK cells produced *in vitro* but not in donor NK cells. In contrast, NKG2D was detected only in donor NK cells but not in *in vitro*-produced iNK cells. iNK cells generated using Protocol 2 expressed the highest level of senescence-related proteins CD57, TIM3 and TIGIT that correlated with its poor performance for yield and purity, prompting us to exclude this protocol from further characterisation.

To further characterize iNK cell identity at the transcriptional level, we compared their gene expression profiles with those of UCB and PB-derived CD34-positive progenitor cells, PBMCs, PBMC-NK, and the iPSCs starting material, using scRNAseq (Supplementary Figure S3; Supplementary Table S3). As shown in Figure 2Bi, iNK cells generated using Protocols 3 and 7 exhibited distinct transcriptional profiles that clearly segregated them from the other analysed cell types. Focused analysis of transcription factors and functional genes associated with NK cell specification demonstrated a pronounced enrichment of canonical NK cell markers in iNK populations (Figure 2Bii). Moreover, transcripts encoding proteins directly involved in pro-inflammatory and cytotoxic effector functions were highly enriched in iNK cells (Figure 2Biii).

**FIGURE 3:**
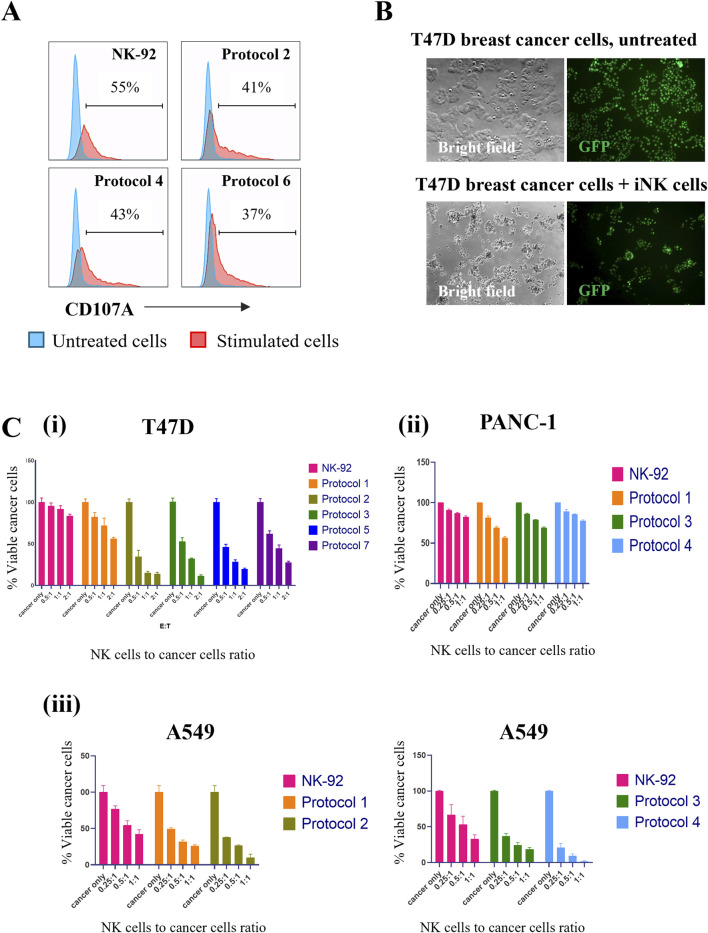
Cytotoxic potential of iNK cells obtained by different protocols. The cytotoxic activity of iNK cells was tested on multiple cancer cell lines and compared to the activity of NK92. **(A)** Translocation of CD107A upon activation. iNK cells or NK-92 cells were co-cultured with Raji cells at 1:1 ratio for 2 h in the presence of CD107A Ab and stained for CD56. **(B)** Phase contrast and fluorescent images of T47D cells (breast cancer cell line) expressing GFP reporter before and after 48-h co-culture with iNK cells differentiated by Protocol 3. **(C)** Plots showing the reduction of adherent cancer (Target, T) cell numbers after co-culture with iNK (Effector, E) cells for 48 h at E:T ratio 0:1, 0.25:1, 0.5:1 and 1:1. iNK cells were differentiated by various protocols for 40 days and added to adherent cancer cells. Image-based data collection was performed for T47D, breast cancer cells expressing GFP reporter (i) and PANC-1, pancreatic cancer cell line expressing NIR FP reporter (ii); luciferase-based cytotoxicity assay targeting A549, lung cancer cells, modified with luciferase reporter showed dose dependent killing (iii). For all cytotoxicity studies, if it was not specifically identified, the iNK cells were collected from cultures at Day 40 of differentiation from iPSCs.

High level of CD56 expression at both the protein and transcriptional levels indicates that *in vitro* -produced iNK cells acquire a “CD56 bright” phenotype. However, unlike donor-derived “CD56 bright” NK cells that are typically less cytotoxic, the *in vitro*-produced iNK cells exhibit high expression of cytotoxicity-related genes including *PRF1* (perforin), *GZMA* and *GZMB* (granzymes) and NKG7, a regulator of cytotoxic granule exocytosis. The expression of these cytotoxicity-related genes was generally higher in iNK cells generated using Protocol 3 compared to Protocol 7, which was reflected in their enhanced ability to kill cancer cells (Figure 3Ci).

While iNKs generated with either protocol in static 2D conditions show clear upregulation of NK lineage markers and the cytotoxicity machinery, they also display a high proliferative potential (by upregulation of CCND2, Cyclin D2, and BCL1 (an anti-apoptotic protein), active Notch signalling (MAML3, (Oyama et al., 2011)) and promiscuous T cell fate commitment (THEMIS), suggesting that the differentiation protocols are driving a functional but distinct phenotype.

Collectively, the combined phenotypic and transcriptional data indicate that the *in vitro* generated iNK cells display a mature NK cell profile and are functionally equipped for effective cancer cell killing.

### 3.4 Mature iNK cells are cytotoxic against cancer cells

We first assessed whether iNK cells can recognise and be activated by cancer cells. CD107A(LAMP-1) is a marker of NK cell degranulation, which surface expression is upregulated upon contact with target cancer cells and correlates with the cytotoxic granule release by NK cells. As shown in Figure 3A, CD107A is upregulated on iNK cells following brief co-incubation with Raji leukemia cells. The human NK-92 cell line, the immortalised NK cells earlier proposed as an off-the-shelf therapy for immuno-oncology applications, was used as a control in this assay.

To further evaluate the ability of iNK cells to recognize and eliminate malignant cells, a series of co-culture assays was established in which iNK cells were co-cultured with various adherent cancer cell lines for time periods of up to 48 h. Cancer cells expressed reporter genes, such as luciferase or fluorescent proteins, enabling quantitative assessment of cancer cell survival following exposure to iNK cells. An example of iNK-mediated cytotoxicity is shown in Figure 3B using a GFP-expressing breast cancer cell line, where a marked reduction in fluorescent cell numbers and pronounced morphological deformation observed in bright-field images indicated effective cytotoxic activity.

Using high-throughput screening approaches, the cytotoxic activity of iNK cells generated using several differentiation protocols was quantified against multiple cancer cell lines. Significant cytotoxic effects were observed at an E:T ratio of 0.25:1, with rapidly increasing cytotoxicity at higher ratios of 0.5:1 and 1:1. As shown in Figure 3C, iNK cells exhibited superior, dose-dependent cytotoxic activity against breast, pancreatic, and lung cancer cell lines compared with NK-92 cells. Notably, differential sensitivity among cancer cell lines to iNK-mediated killing was observed, which may reflect tumor-specific immune evasion mechanisms, including expression of inhibitory surface ligands or secretion of immunosuppressive factors such as transforming growth factor-β (TGF-β) or prostaglandins.

To further characterize activation responses, iNK cells were co-cultured for 24 h with K562 cells, a potent NK cell activator, at an E:T ratio of 1:2. UCB-NK cells were used as controls. Following co-incubation, NK cells were collected and analyzed by multiparametric flow cytometry to assess changes in the expression of activating and inhibitory receptors associated with NK cell cytotoxic responses. Baseline expression levels were determined using unstimulated NK cells.

iNK cells exhibited higher surface expression of CD69, an early activation marker, both at baseline and following activation with K562 cells compared with UCB-NK cells (Figure 2A; Supplementary Figure S4). Although expression of DNAM-1, an activating adhesion receptor, was lower on iNK cells relative to UCB-NK cells under both baseline and stimulated conditions, the frequency of CD69/DNAM-1-double positive cells remained high, indicating preservation of cytotoxic competence. Overall, iNK cells displayed a more pronounced activation phenotype, characterized by elevated basal activation and a robust response upon challenge.

**FIGURE 4:**
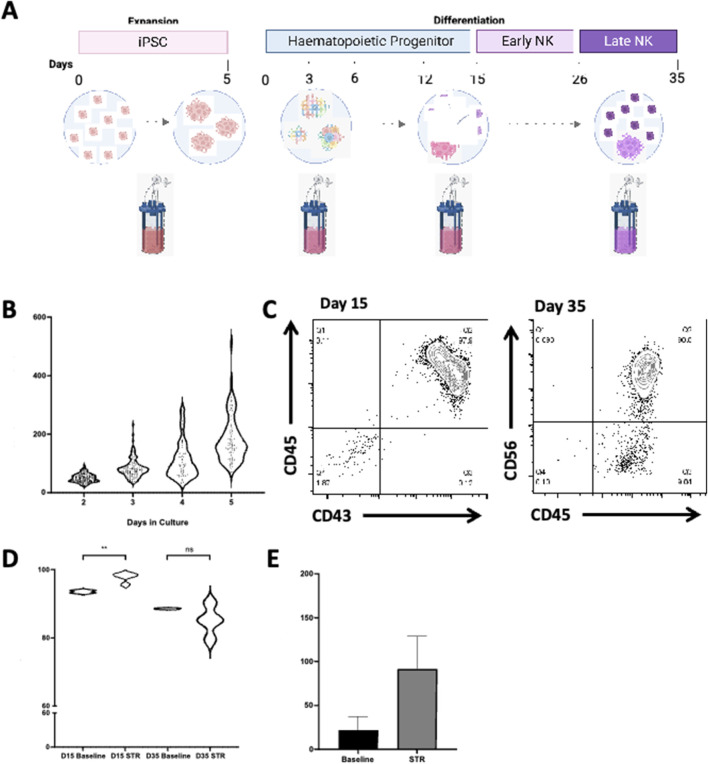
Manufacturing of iNK cells in STR bioreactor. **(A)** iPSC to NK differentiation protocol. Seamless expansion and differentiation of iPSC in a stirred tank bioreactor incorporating a 5-day expansion of iPSC from single cells into aggregates, and differentiation into NK cells over 35 days in the same system. **(B)** Aggregate diameter measured from day 2 to day 5 of iPSC expanded in a stirred tank bioreactor. Dashed line (---) represents mean and crosses (x) are individual aggregate diameters. At least 50 aggregates analysed per day (range 50–233). **(C)** Target cell phenotype measured by flow cytometry. CD43 and CD45 used for haematopoietic progenitor identification on day 15, and CD45 and CD56 used to identify NK cells on day 35. Samples taken from four vessels at each timepoint (n = 4). **(D)** Comparison of target cells at day 15 and day 35 produced by the static process and in a stirred tank bioreactor (n = 4). **(E)** Process yield calculated as the number of NK cells produced per starting iPSC across conditions (baseline 2D process (n = 3), STR differentiation (n = 4)). Data presented as mean + standard deviation.

Concurrently, iNK cells expressed lower levels of TIGIT, a marker associated with NK cell exhaustion, both at baseline and after activation compared with UCB-NK cells (Supplementary Figure S2A; Figure 4).

In contrast, expression of the inhibitory receptor TIM-3 was slightly higher on iNK cells under both conditions relative to CB-NK cells. Importantly, iNK cells maintained high expression of activating and cytotoxic molecules, including NKp46, CD69, and perforin, and did not exhibit significant upregulation of exhaustion-associated markers such as CD57 or TIGIT following stimulation with K562 cells (Figure 2A; Supplementary Figure S4).

It is important to note, that iNK cells selectively targeted cancer but not normal cells, as evidenced from the increased death rate in K562 and Raji cells after brief 3 h co-culture with iNK cells, while healthy PBMC cells remained intact (Supplementary Figure S5). This suggests low off-target cytotoxicity of iNK cells, which defines a good safety profile, although further quality control tests will be needed.

In summary, these data demonstrate that iNK cells display a distinct activation profile characterized by elevated basal and inducible activation, a critical feature that allows them to act as a rapid, first-line defence against tumour cells. Together with their superior cytotoxic activity, these properties support the potential of iNK cells as efficacious therapeutic candidates for cancer immunotherapy.

### 3.5 Adapting the CombiCult®-derived protocol to iNK cell production in an STR

For scalable generation of clinically relevant numbers of iNK cells, transition of the differentiation Protocol 7 to STRs was attempted (Figure 4A). Parameters for iPSC culture in STRs were optimised over two runs to generate consistent starting material for subsequent differentiation to iNK cells. Seeding density, perfusion rate, and agitation rate were assessed with cultures initiated from iPSC dissociated into single cells. Once optimised, cells expanded robustly to achieve 1.2 × 10^9^ cells in 190 mL of culture volume with >80% viability, once dissociated and counted. Reduction in iPSC numbers was observed after 24 h however doubling times settled between 20 and 30 h from 24–120 h of culture. Glucose depletion was observed by 72 h, and this was stabilised through glucose supplementation of the cell culture medium. Aggregate size increased from ∼50 µm after 24 h to ∼200 µm by 120 h (Figure 4B), and flow cytometry confirmed maintenance of pluripotency markers with >80% expression of surface and intracellular markers post-dissociation into single cells.

**FIGURE 5:**
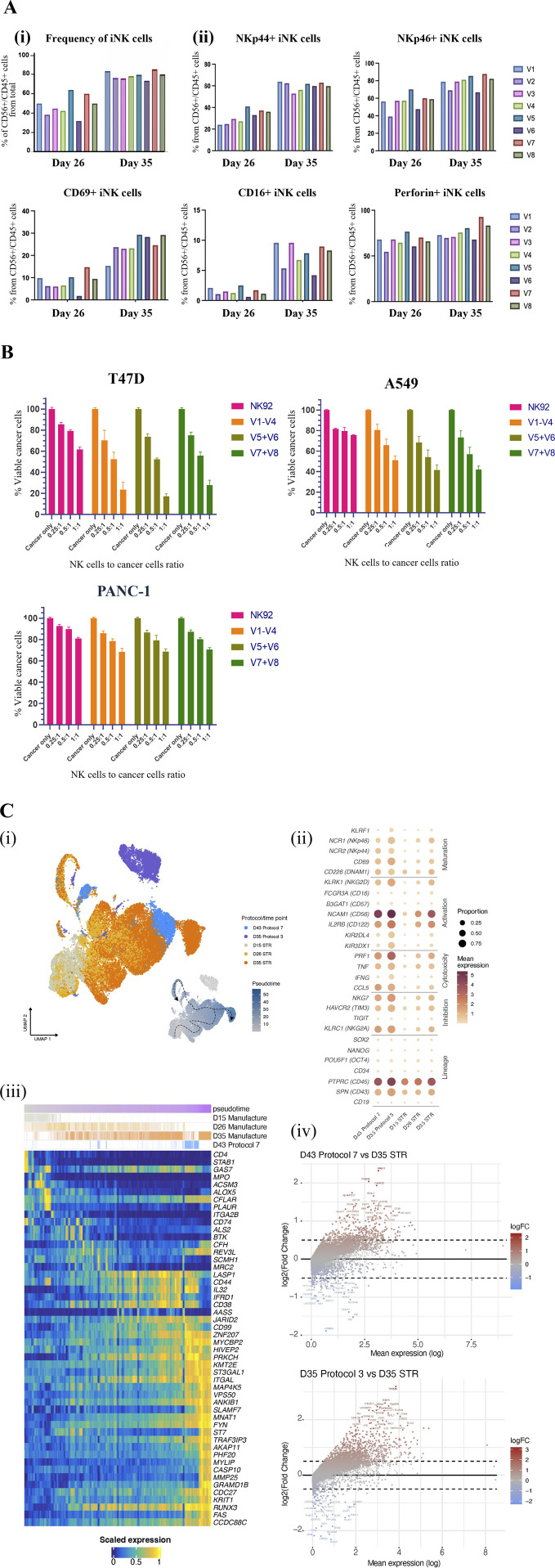
Characterization of iNK cells produced in STR bioreactor. iNK cells were collected at Days 26 and 35 from an STR bioreactor run using Protocol 7. Four manufacturing conditions were tested in duplicate vessels per condition (altogether 8 vessels, V1 ÷ V8). **(A)** (i) iNK cell frequency is steadily increased in the production vessels by Day 35. (ii) Multiparametric flow cytometry analysis showing profile of surface and internal molecules expressed by iNK cells produced in STR. **(B)** iNK cells produced by manufacturing runs are highly cytotoxic. Cells from each vessel were analyzed separately and results grouped by manufacturing condition. Image-based data collection was performed for T47D GFP-expressing breast cancer cells and PANC-1 pancreatic cancer cell line expressing NIR FP reporter; luciferase-based NK killing assay targeting A549 modified with luciferase reporter showed dose dependent killing of lung cancer cells. **(C)** (i) UMAP embedding of the single cell transcriptomes for static conditions and STR-produced conditions at Days 15, 26, and 35. Inset: monocle pseudotime estimation starting from day D5 cells. (ii) Dot plot showing the mean log-normalized expression and percentage of expressing cells of genes relevant for different aspects of NK differentiation and function. (iii) Heatmap of genes that are differentially expressed along the pseudotemporal trajectory. (iv) Mean-difference plots showing differential expression (log2(fold change), y-axis) and mean normalized expression (x-axis) of genes according to the Wilcoxon rank sum test comparing Day 43 Protocol 7 iNKs (upper panel) and Day 35 Protocol 3 iNKs (lower panel) to Day 35 STR-produced iNKs.

A complete medium exchange was performed in the STR to initiate differentiation to NK cells while the static control was maintained according to the baseline protocol in 6-well plates. Cells remained within aggregates until Day 12 of differentiation with cell number in the aggregates steadily increasing during that time. From Day 12 onwards single cells emerged from aggregates into suspension characterised by expression of CD34, CD43 and CD45, with cells positive for CD45 and CD43 comprising ∼98% of cells in suspension in STR cultures as opposed to ∼93% in static cultures (p < 0.01, Figures 4C,D) suggesting the controlled culture environment in STRs supports more consistent and efficient differentiation to haematopoietic progenitor cells compared to static culture. Morphologically, cystic aggregates were observed from Day 6 onwards with the emergence of bright, rounded cells from Day 12. Cells in suspension increased consistently throughout differentiation in the STR whereas cell number plateaued Day 26 onwards for the baseline protocol. iNK cells were first observed on Day 26 with ∼40% expression of CD56/CD45-double positive and rising to >80% CD56/CD45-double positive by Day 35 (Figures 4D, 5Ai). Process yield was increased in the STR from ∼20 iNK cells generated per iPSC in the baseline process to ∼190 iNK cells in the STR process (Figure 4E).

### 3.6 iNK cells produced in STR are mature and functional

We aimed to determine the maturation status of suspension iNK cells generated during STR manufacturing runs by analysing several key surface and intracellular markers that define iNK cell maturation and activation potential. While iNK cells harvested at Day 26 displayed an immature phenotype and insufficient iNK cell purity (Figure 5Ai), the continuous STR culture until termination (Day 35) became enriched in CD56/CD45-double positive population with enhanced expression of key NK activation receptors, including NKp44, NKp46, and CD69. Although iNK cells are often reported to lack CD16 expression, we observed that 5%–10% of STR-produced iNK cells were CD16-positive (Figure 5Aii). Notably, expression of these markers, together with intracellular perforin, was higher in iNK cells generated using Protocol 7 under static 2D conditions at differentiation Day 42 (Figure 2Ai), suggesting that further extension of the production timeline may improve both purity and maturation status. Nevertheless, iNK cells differentiated in STR demonstrated robust cytotoxic potential against cancer cell lines of diverse origins in previously established assays (Figure 5B). STR-produced iNKs at Days 15, 26, and 35 were also profiled by scRNAseq, and compared to data from cells produced in static 2D conditions following Protocols 3 and 7 (Figure 5C; Supplementary Table S4). While Protocol 7 is embedded in a continuum with the manufactured iNKs, Protocol 3 forms its own distinct cluster, potentially owing to differences in differentiation and maintenance media (Figure 5Ci; Supplementary Table S5). The expression of markers of activation, maturation and cytotoxicity increases over time and reaches levels comparable to those observed in static 2D conditions (Figure 5Cii). Estimating a pseudo-temporal trajectory starting from Day 15 STR cultures, stepwise expression of NK developmental markers can be observed, such as a downregulation of *MPO*, transient upregulation of *BTK,* and sustained expression of *CD38* (Figure 5Ciii). Moreover, differential expression between static 2D and STR-produced protocols at their endpoints (Day 43 Protocol 7 vs. Day 35 STR, Figure 5Civ) highlights important molecular differences such as increase in *GNLY*, *CCL5* (RANTES)*, PRF1* (Perforin) in the static 2D conditions compared to the STR-produced ones, while conversely *RUNX1*, *TOX2* are more highly expressed in manufactured iNKs. These results agree with what was observed by flow cytometry, suggesting a longer culture in STR can further increase the activation and maturation of iNKs.

## 4 Discussion

The translation of manually optimized bench-scale differentiation protocols into scalable manufacturing remains one of the challenges in the development of cell-based immunotherapies. Although iPSC to NK differentiation protocols exist, the published protocols rely on the use of feeder cells, animal-derived coatings, and/or serum (Hermanson et al., 2015; Zhu and Kaufman, 2019; Euchner et al., 2021). In this study, we applied a combinatorial screening strategy within a 3D culture system to identify efficient and manufacturing-compatible protocols for generating iNK cells under feeder-free and/or serum-free condition. The 3D setup that more closely resembles scalable production conditions helped us to bridge the gap between early protocol development and clinical-scale manufacturing.

The combinatorial screen enabled systematic identification of differentiation conditions that promoted differentiation of iPSCs into of CD56/CD45 double positive iNK cells. This approach yielded iNK cells displaying a broad spectrum of phenotypic characteristics and cytotoxic capabilities. Such diversity is valuable in the context of anti-cancer therapy, as distinct NK phenotypes may differ in persistence, activation status, cytotoxic potential, and resistance to tumor-induced suppression. The ability to select differentiation protocols based on predefined functional and phenotypic criteria represents a significant advantage for manufacturing a therapeutic product with tailored anti-tumor properties.

A critical technical aspect of the CombiCult® platform is the use of split-pooling and tagging procedures that require cell encapsulation in beads. Consistent with previous observations from megakaryocyte differentiation (Tarunina et al., 2026), when iPSCs were encapsulated in alginate beads as a single-cell suspension they failed to efficiently differentiate into hematopoietic lineages while encapsulation of iPSC aggregates enabled hematopoietic specification. This highlights the importance of cell–cell interactions and microenvironmental cues during early lineage commitment. The use of aggregates in alginate beads resembles cellular aggregate-based culture employed in STR manufacturing. Therefore, selection of differentiation conditions directly within a 3D aggregate context increases translational relevance and reduces the need for subsequent process optimization.

Two distinct NK cell populations emerged from the screen. iNK cells differentiated using Protocol 7 were characterized by CD16 expression in approximately 10%–20% of the cell population, along with elevated expression of activation markers CD69 and DNAM-1 as determined by both FACS and scRNAseq analysis. scRNAseq also revealed increased expression of transcription factor *IKZF3*, a member of the Ikaros family of zinc-finger transcription factors, that has been reported to play an important role in the regulation of NK cell maturation, cytotoxic function, and interferon-γ secretion (Holmes et al., 2014). iNK cells generated by this protocol demonstrated functional cytotoxicity against cancer cell lines. Although the presence of serum in the final differentiation medium of Protocol 7 makes this protocol less suitable for future clinical applications, it proved valuable for establishing and optimizing STR-based manufacturing due to its relative simplicity and cost-effectiveness.

iNK cells generated using serum-free feeder-free Protocol 3 demonstrated enhanced cytotoxicity and elevated expression of cytotoxicity-associated markers, including *NKG7*, perforin and granzyme B, along with reduced expression of exhaustion and senescence markers such as TIGIT compared to PB-NK cells. Notably, these cells exhibited higher expression of *LEF1*, a key transcription factor in NKT cells that drive a central memory, promoting expansion, longevity, and reducing cell exhaustion (Ngai et al., 2023). scRNAseq analysis revealed that iNK cells generated by Protocol 3 and at slightly less degree by Protocol 7 exhibited markedly higher expression of the transcription factor *ZNF683* compared to PB-NK cells. ZNF683+ NK cells have been implicated in modulating the tumor microenvironment through interaction with CD8^+^ T cells potentially preventing tumor chemoresistance (Li et al., 2026). Collectively, these features suggest a potentially superior functional profile for anti-tumor applications.

Although serum-free, feeder-free protocols may be more complex in terms of the factors required, they offer the advantage of being precisely defined resulting in improved reproducibility and reduced susceptibility to batch-to-batch variation and xenogeneic contaminants. Furthermore, such systems, which rely on defined components, are more compatible with GMP requirements and closed, fully automated bioreactor platforms. However, Protocol 3 includes DLL4 at multiple stages of differentiation, significantly increasing production costs. Further optimization will therefore be necessary to simplify media composition, reduce reliance on costly components, and determine whether comparable functional outcomes can be achieved under more economical conditions.

Successful translation of 3D CombiCult®-derived Protocol 7 into manufacturing was achieved within two experimental STR runs. We attribute rapid and successful transfer of manual protocols into manufacturing process to the fact that the optimal conditions for the 3D culture/differentiation were selected during alginate-beads based combinatorial screen. Cell culture parameters including initial cell density, starting aggregate number and frequency of media change were directly replicated from manual process while other parameters such as media dilution factor had to be adjusted to avoid glucose depletion and lactate accumulation both of which resulted from enhanced proliferation observed under bioreactor conditions. Transfer of manual protocols into STR manufacturing led to approximately 10x increase in efficiency of differentiation producing ∼190 iNK cells per iPSC compared to 20 iNK per iPSC in the original manual process. This increase in iNK yield is likely attributable to (1) more uniform starting material generated in the agitated and perfused bioreactors, producing more homogeneously sized iPSCs aggregates, (2) controlled culture environment with dilution and media refreshment, (3) faster shedding of emerging hematopoietic cells. iNK cell populations produced by Protocol 7 under manual and manufacturing conditions exhibited very similar properties in terms of activation, inhibitory and exhaustion marker expression, cytotoxicity against cancer cells and gene expression profile.

Several limitations should be acknowledged. While phenotypic and cytotoxic profiles were characterized, additional *in vivo* studies are required to assess persistence, safety, and anti-tumor efficacy. Moreover, long-term stability, scalability under bioreactor conditions, and batch-to-batch consistency must be evaluated before clinical translation. Finally, a more detailed molecular characterization of the two NK subsets may provide insight into mechanisms underlying their functional differences and inform rational process refinement.

## 5 Conclusion

Our study demonstrates that combinatorial screening in a 3D aggregate-based system is a powerful strategy for identifying manufacturing-compatible protocols for iNK cell production. By integrating phenotypic, functional, and cost considerations at the early stages of protocol selection, this approach supports the development of scalable and clinically relevant NK cell therapies. Further refinement of media composition and functional validation will be essential to fully realize the translational potential of this platform.

## Data Availability

The sequencing data that support the scRNAseq findings in this study have been deposited to Gene Expression Omnibus (GEO), GEO accession number: GSE323336.
