# Supplementary material for: From iPSC to manufactured iNK cells using CombiCult® screening platform
Source: Front Cell Dev Biol. 2026 Jun 11;14:1824021. doi: 10.3389/fcell.2026.1824021 (PMC13294436; doi:10.3389/fcell.2026.1824021)
Supplement: Supplementary file 3 [file Supplementaryfile1.pdf]

From iPSC to manufactured iNK cells using CombiCult® screening platform

Marina Tarunina[a*], Sachin Luharia[a], Matthew Houppermans[a], Giuseppe D'Agostino[a], Lam Lam[a], Michelle Gestwa[a], Aleksandra Habich-Crayton[a], Marcia Mata[c], Juline Guenat[c], Molly Tregidgo[c], Patrick Statham[c], Charlotte Lee-Reeves[c], Mudith Jayawardena[c], Simona Zingaro[c], Limor Zwi-Dantsis[c], Aishwarya Nair[c], Alexandru-Robert Podovei[c], Vera Karels[c], Jahid Hasan[c], Tatyana Ponomaryov[a], and Yen Choo[a,b,*]

[a]Plasticell Ltd., Stevenage Bioscience Catalyst, Gunnels Wood Road, Stevenage SG1 2FX, UK.
[b]Cancer Discovery & Regenerative Medicine, Lee Kong Chian School of Medicine, 11 Mandalay Road, Singapore
[c]Cell and Gene Therapy Catapult, Guy's Hospital, Great Maze Pond, London SE1 9RT, UK

[*]Authors for correspondence and reprint requests:

Dr Marina Tarunina
marina@plasticell.co.uk

Dr Yen Choo
yen.choo@ntu.edu.sg

Plasticell Ltd
Stevenage Bioscience Catalyst
Gunnels Wood Road
Stevenage
SG1 2FX
United Kingdom
Phone: 01 438 906 906

SUPPLEMENTARY MATERIAL

SUPPLEMENTARY FIGURES

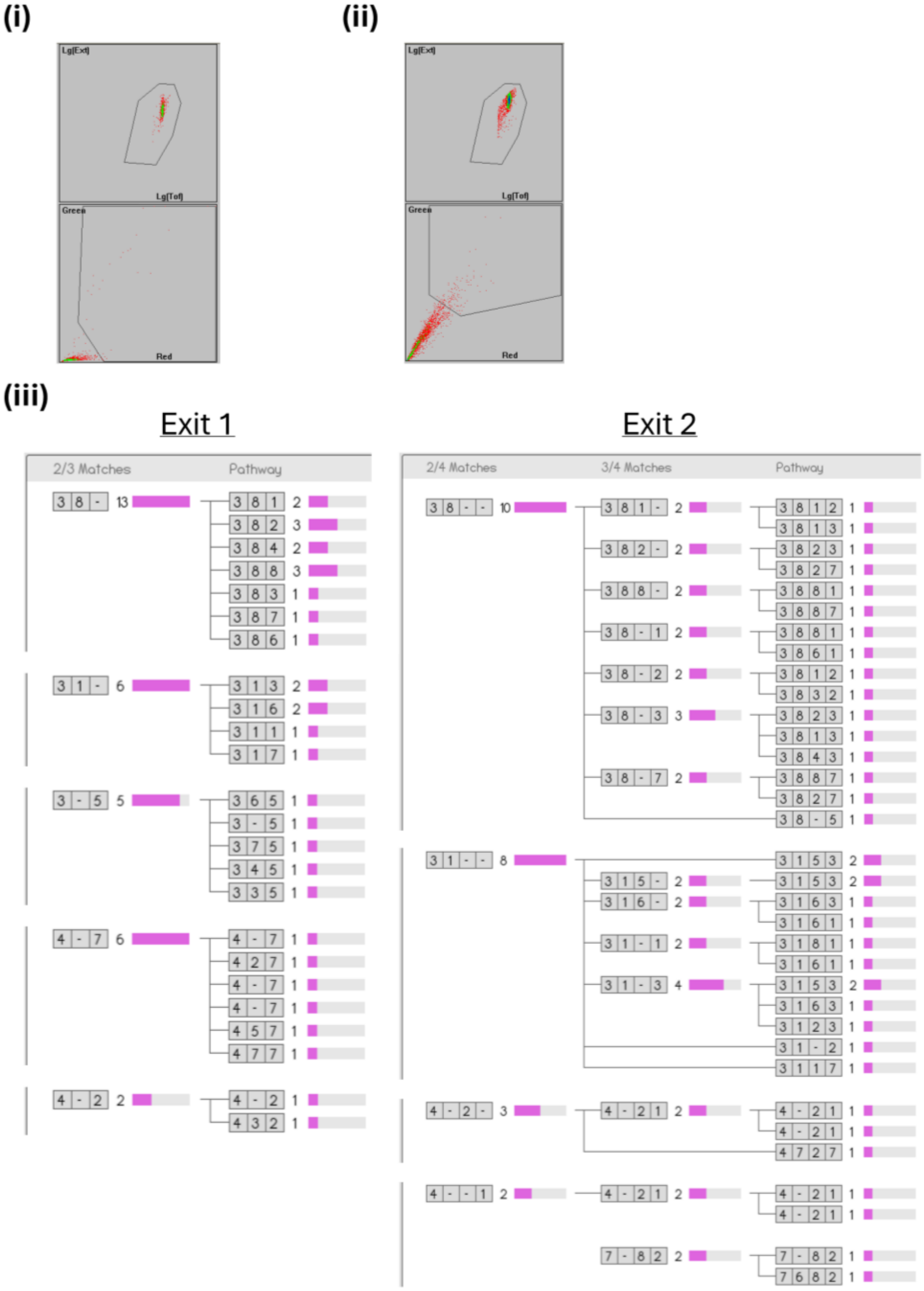

Supplementary Figure 1: COPAS sorting of CombiCult® Exit 1 and Exit 2 positive beads and identification of hit protocols by Ariadne™ analysis.

(i) Exit 1: Single beads containing CD45/EdU click positive cells were first gated based on size (X axis) and optical density (Y axis) (upper plot) and then sorted based on the intensity of red (CD45+) and green (EdU click) signal (bottom plot).

(ii) Exit 2: Single beads containing CD45/CD56 positive cells were first gated based on the size (X axis) and the shape (Y axis) (upper plot) and then sorted based on the intensity of red (CD45+) and green (CD56+) signal (bottom plot).

(iii) Ariadne clustering analysis enabled selection of best hit protocols for further validation. Example: media sequence 3-8-2 received highest score in Exit 1 and was represented by multiple beads (triplets) and big cluster. Protocol cluster 3-8-X-3 was prominent in Exit 2 leading to selection of Protocol 4 (3-8-2-3) for further validation.

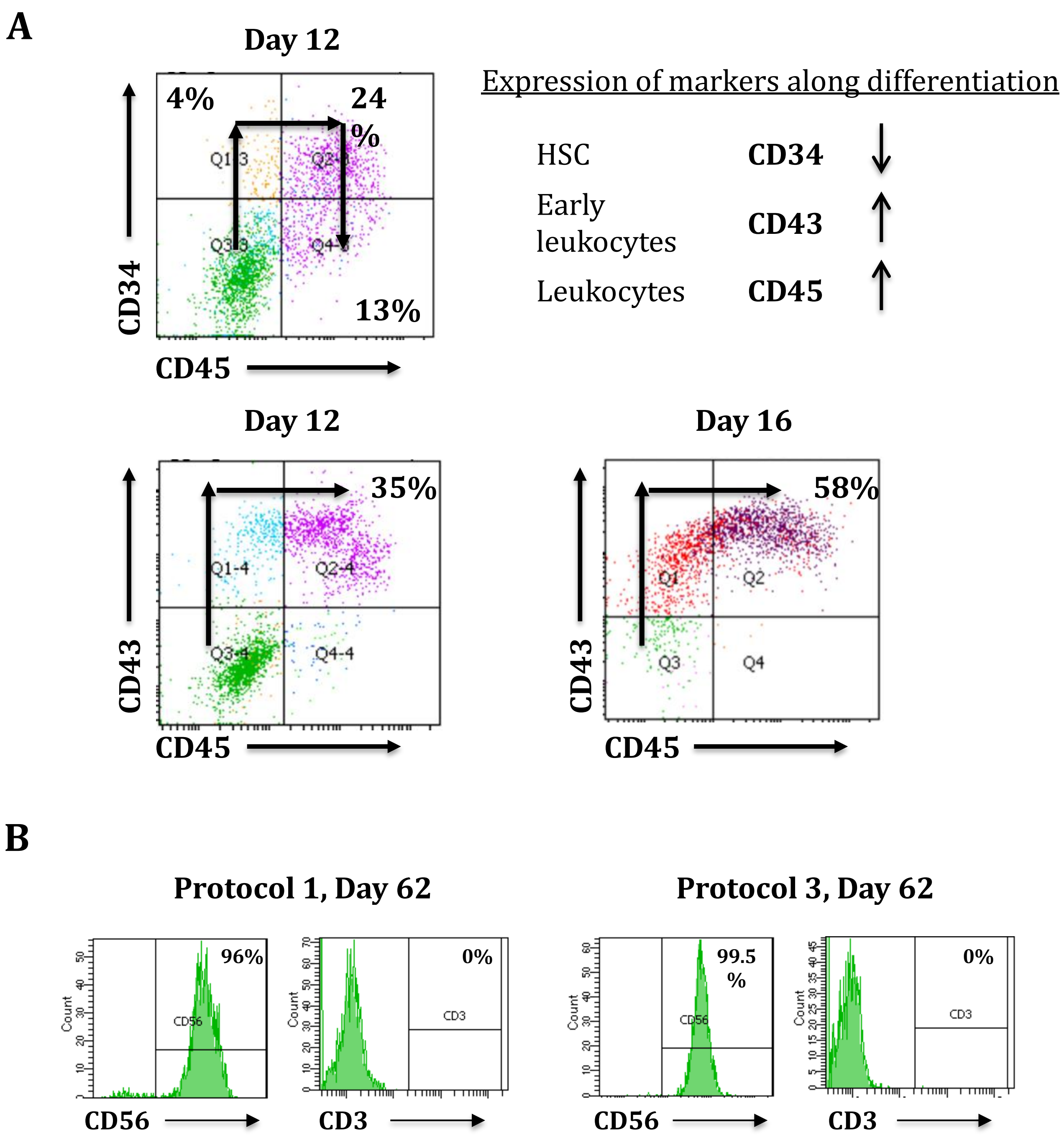

Supplementary Figure 2: Expression of hematopoietic and maturation markers following iNK cells differentiation in Static 2D cell cultures.

A. The accumulated hematopoietic CD34-pos cells slowly lose CD34 marker expression. Along the differentiation process towards leukocytes, the CD43 marker is expressed earlier, which is further accompanied by appearance of CD45 pan-leukocyte antigen, expressed by more mature cells.

B. Highly pure CD56 NK cells develop in the long-term cultures without contamination with CD3-positive T cells.

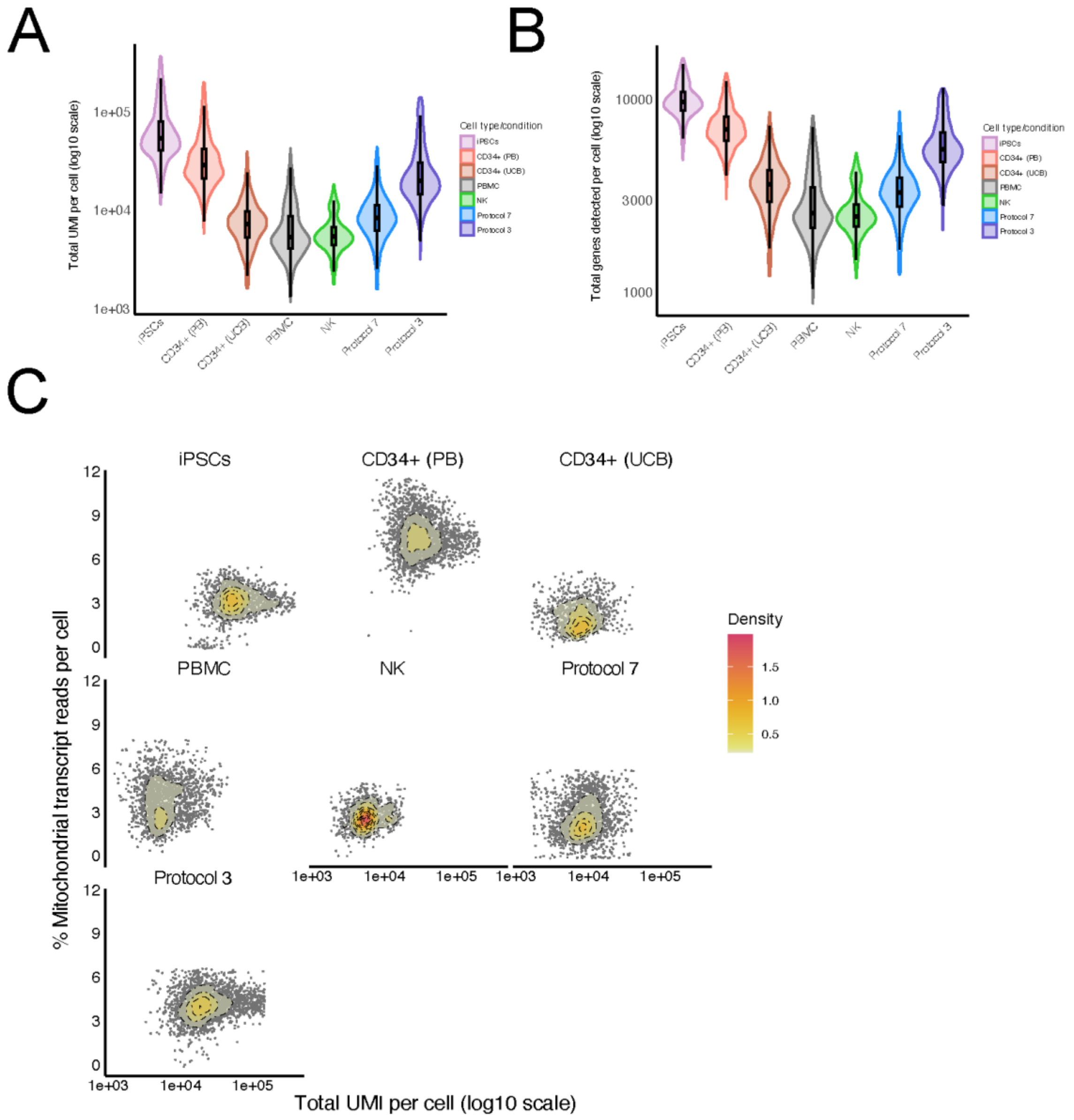

Supplementary Figure 3: Quality Control metrics for the scRNA-seq dataset.

A. Total reads (UMI) per cell grouped by condition/protocol.

B. Total genes detected per cell grouped by condition/protocol.

C. Scatter plots of the percentage of mitochondrial transcripts and the corresponding total reads per cell grouped by condition/protocol

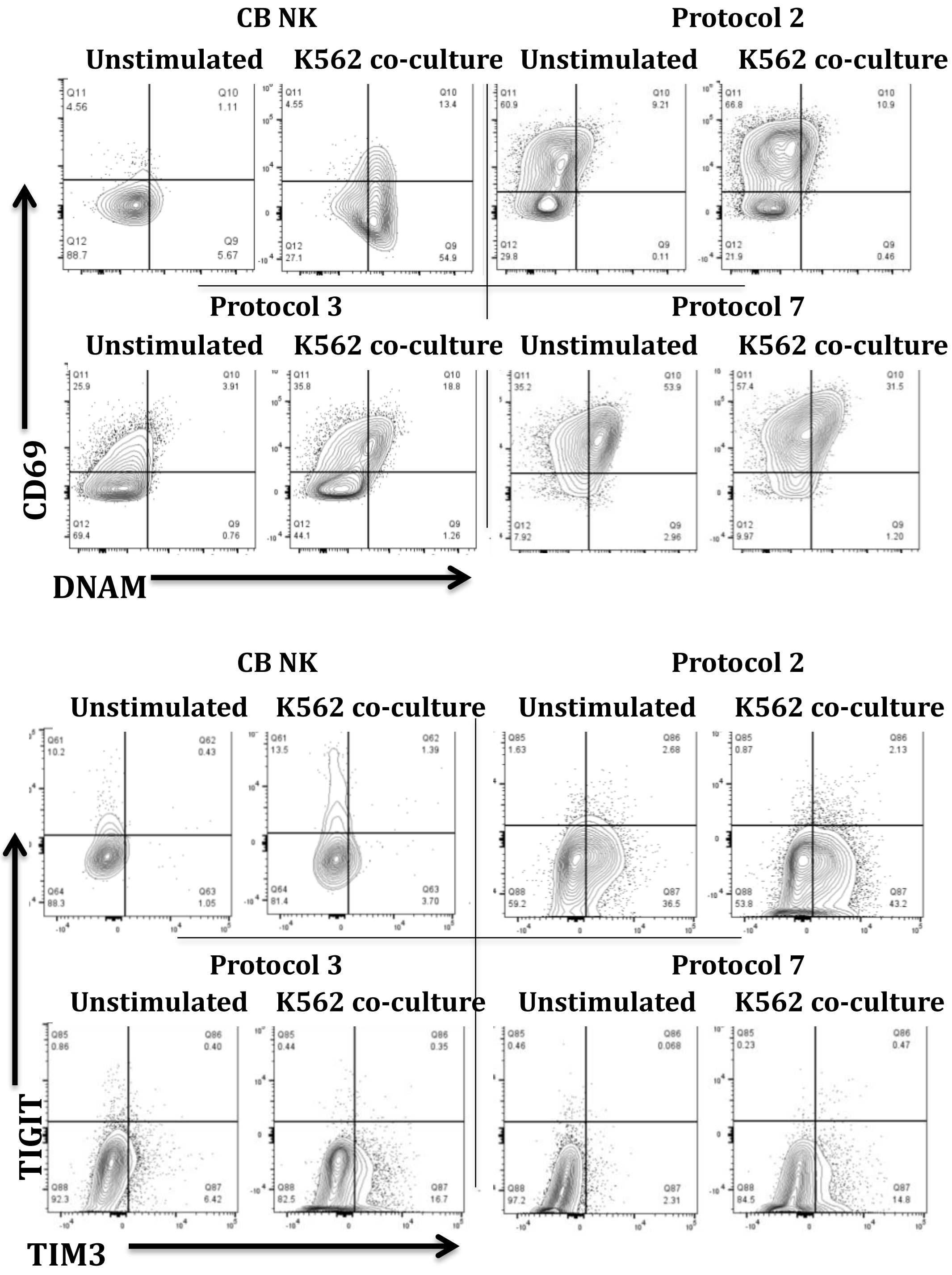

Supplementary Figure 4: Dynamic expression of activating and inhibitory receptors on populations of iNK cells after stimulation with K562 cells.

FACS plots show populations of iNK cells before and after 24 hrs co-culture with K562 cells. The elevated expression of CD69 on iNK cells suggests their high cytotoxic potential, which is not exhausted after exposure to K562 cells. Low or moderate expression of TIGIT and TIM3 on iNK cells suggest that iNK cells might have a prolonged cytotoxic activity. iNK cells were collected at Day 49 (Protocols 2 and 3) and Day 42 (Protocol 7) of differentiation. All measurements were compared to UCB-NK.

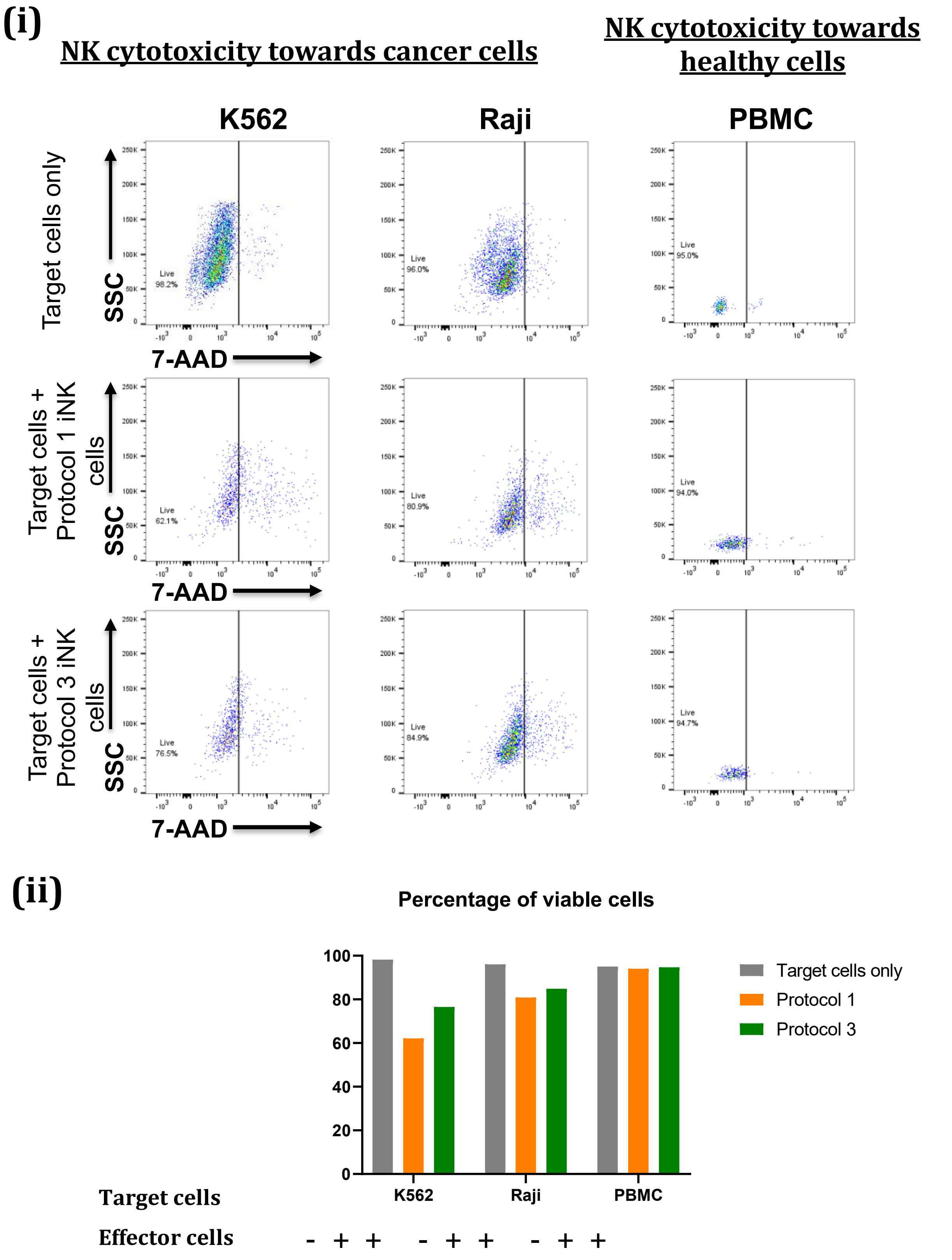

Supplementary Figure 5: Low off-target cytotoxicity of iNK cells.

(i). iNK cells were incubated with K562, Raji or PBMC from a healthy donor at 1:1 ratio for 3 hours. Cytotoxicity was assessed by flow cytometry using 7-AAD as a live/dead stain.

(ii). The increase in percentage of dead cells is plotted for the given experiment.

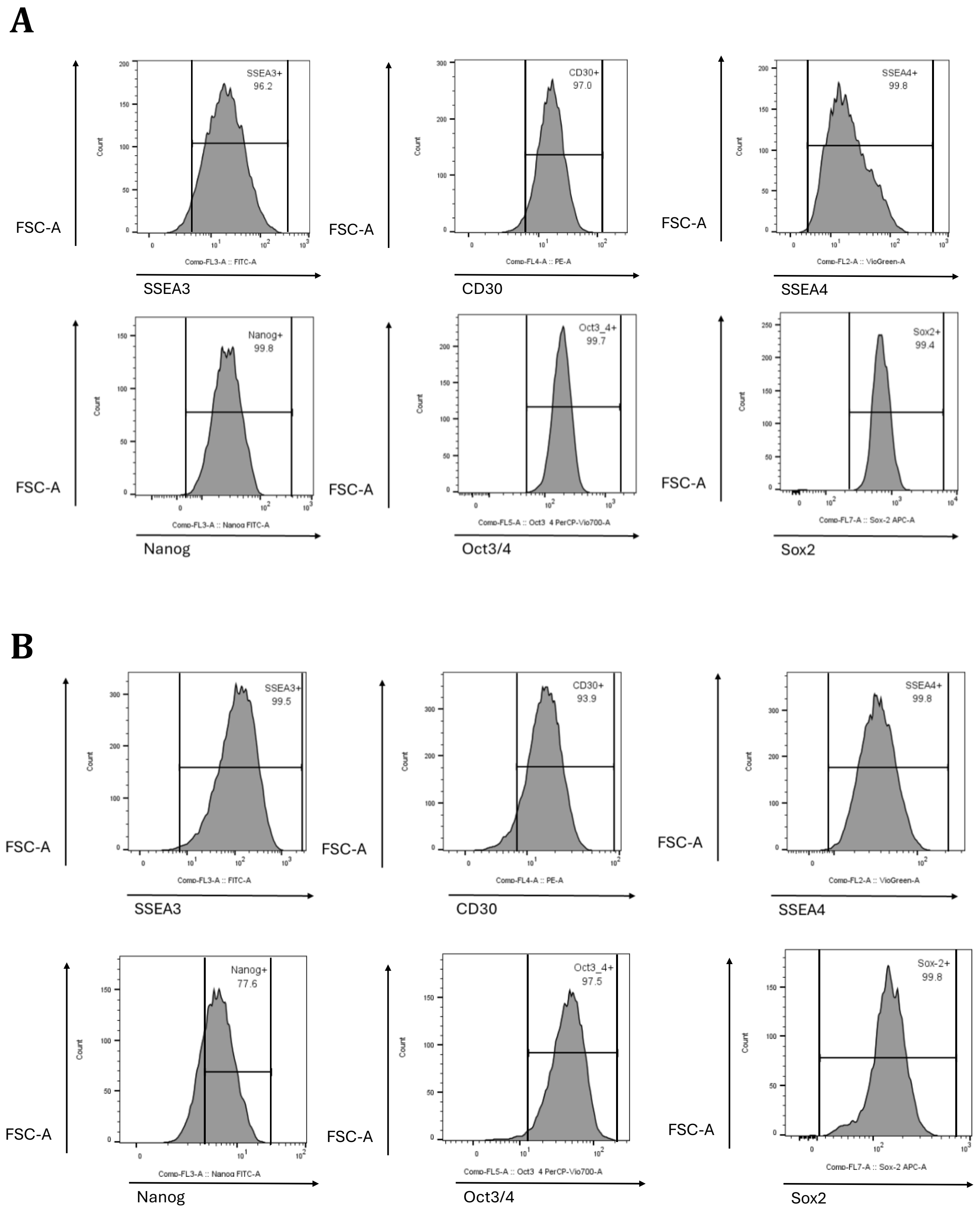

Supplementary Figure 6: Quality control of iPSC in the manufacturing process.

A.Pluripotency by flow cytometry for iPSC prior to inoculation in stirred tank bioreactor system.

B. Pluripotency by flow cytometry of iPSC after 5 days of culture as clusters in stirred tank bioreactor.

Supplementary Methods

1. CombiCult® screening experiment.

Alginate encapsulation.

Alginate beads containing cell clumps were generated using an electrostatically-driven microencapsulator (Nisco). Briefly, mechanically harvested cell clumps were resuspended in a 2.0% (w/v) solution of sodium alginate in PBS. The suspension was dispensed through the nozzle of the microencapsulator at a constant flow rate using a syringe pump. The droplets generated by this procedure were crosslinked into alginate beads by means of ionotropic gelling in a 200 mM calcium chloride (Sigma-Aldrich, C5670) solution. The resulting alginate beads were washed twice in DMEM before being resuspended in differentiation media and placed in an incubator at 37°C with a humidified atmosphere of 5% CO_2. The alginate beads formed ranged in size from 400–500µm. Under the conditions used, 1ml of alginate produced ~8,000 beads, averaging 300 cells/bead.

Split-pool.

Beads were divided between 8 media conditions for Day 0 (Suppl. Table 1) and plated into compartmentalized 100mm Square Petri Dishes (Thermo Scientific, 103). Beads were incubated for 3 days in the first set of conditions, after which the beads from each condition were tagged with a unique fluorescent tag using a modified alginate multilayering technique (Tarunina M 2019). The beads were then pooled and split into 9 new conditions on Day 3. This split-pool cycle was repeated on Day 6 when beads were tagged with new set of fluorescent tags, pooled and split into 8 new conditions. On Day 10, part of the beads was incubated with EdU. On Day 12, beads incubated with EdU were collected and fixed for Exit 1, while the rest of the beads were tagged, pooled and split between 7 final conditions until Day 32, when all the remaining beads were collected and fixed for Exit 2.

Reagents used in the CombiCult® experiment.

Items	Abbreviation in Supplementary Table 1	Supplier	Catalog number
Media			
Gibco™ DMEM, high glucose, GlutaMAX	DMEM	Gibco	31966021
Gibco™ Ham's F-12 Nutrient Mix, GlutaMAX	F12	Gibco	31765027
STEMdiff™ APEL™2 Medium	APEL2	StemCell Technologies	05275
Proteins			
Human BMP-4 Recombinant Protein	BMP4	Peprotech	120-05
Human CXCL12 (SDF-1a) Recombinant Protein	SDF1	Peprotech	300-28A
Human IL-2 Recombinant Protein	IL-2	Peprotech	200-02
Human IL-3 Recombinant Protein	IL-3	Peprotech	200-03
Human IL-6 Recombinant Protein	IL-6	Peprotech	200-06
Human IL-15 Recombinant Protein	IL-15	Peprotech	200-15
Human IL-21 Recombinant Protein	IL21	Peprotech	200-21

IgG Fc Cross-Adsorbed Goat anti-Human	anti-IgG Ab	Invitrogen	31125
Recombinant Human/Mouse/Rat Activin A Protein	Activin A	R&D Systems	388-AC
Recombinant Human DLL4 Fc Chimera Protein	DLL4 Fc	R&D Systems	10185-D4-050
Recombinant Human FGF basic/FGF2/bFGF (145 aa) Protein	bFGF	R&D Systems	3718-FB
Recombinant Human Flt-3 Ligand/FLT3L Protein	FLT3L	R&D Systems	308-FK
Recombinant Human IGF-I/IGF-1 Protein	IGF-1	R&D Systems	291-G1
Recombinant Human IL-7 Protein	IL-7	R&D Systems	207-IL
Recombinant Human Jagged 1 Fc Chimera Protein	Jagged 1 Fc	R&D Systems	1277-JG
Recombinant Human SCF Protein	SCF	R&D Systems	255-SC
Recombinant Human Thrombopoietin Protein	TPO	R&D Systems	288-TP
Recombinant Human VEGF 165 Protein	VEGF	R&D Systems	293-VE
Biologicals			
β-Estradiol	Estradiol	Tocris	2824
Human IL-15Rα sushi	Sushi	Miltenyi	130-104-914
Human Serum	Serum	Sigma-Aldrich	H6914
L(+)-Ascorbic acid	L-AA	VWR	83568.180
L-Glutamine (200 mM)	L-glutamine	Gibco	25030081
L-Tryptophan	Tryptophan	Sigma-Aldrich	T8941
Nicotinamide	Nicotinamide	Tocris	4106
Penicillin-Streptomycin (5,000 U/mL)	P/S	Gibco	15070063
Retinoic acid	Retinoic acid	Sigma-Aldrich	R2625
Chemicals			
Y-27632 dihydrochloride	ROCKi	Tocris	1254
Lithium chloride	LiCl	Sigma-Aldrich	L4408
β-Mercaptoethanol	B-mercaptoethanol	Sigma-Aldrich	07604
Sodium selenite	Sodium selenite	Sigma-Aldrich	S5261
Ethanolamine	Ethanolamine	Sigma-Aldrich	E0135
Rapamycin	Rapamycin	Sigma-Aldrich	R8781

CHIR 99021	CHIR99021	Tocris	4423
4-Diethylaminobenzaldehyde	DEAB	Sigma-Aldrich	D86256
Pifithrin-μ	Pifithrin	Sigma-Aldrich	P0122
Deferoxamine mesylate salt	DM	Sigma-Aldrich	D9533
SB 431542	SB431542	Tocris	1614
XMU-MP-1 hydrochloride	XMU-MP-1	Sigma-Aldrich	SML2233
3-Isobutyl-1-methylxanthine	IBMX	Sigma-Aldrich	I5879
Valproic acid sodium salt	Valproic Acid	Sigma-Aldrich	P4543
ML 228	ML 228	Tocris	4565
SR1	SR1	Calbiochem	182706
Hydrocortisone	Hydrocortisone	Sigma-Aldrich	H2270
SIS3	SIS3	Calbiochem	566405
For B15			
Polyvinyl alcohol	PVA	EMPROVE® ESSENTIAL	1.41351
Cellastim-S	Cellastim-S	InVitria	777HSA017 S
Optibumin® 20 – Recombinant human albumin	rAlbumin	InVitria	777HSA105
Insulin-Transferrin-Selenium-Ethanolamine (100X)	ITS-X	Gibco	51500056
MEM Non-Essential Amino Acids Solution (100X)	NEAA	Gibco	11140035
SyntheChol® NS0 Supplement	SyntheChol NS0	Sigma-Aldrich	S5442
Progesterone	Progesterone	Sigma-Aldrich	P8783
Corticosterone	Corticosterone	Sigma-Aldrich	27840
Linoleic acid	Linoleic acid	Sigma-Aldrich	L1012
Linolenic acid	Linolenic acid	Sigma-Aldrich	L2376
Retinyl acetate	Retinyl acetate	Sigma-Aldrich	R7882
L-carnitine	L-carnitine	Sigma-Aldrich	C0283
Vitamin B12	Vitamin B12	Sigma-Aldrich	V6629

Immunostaining of cells in alginate beads.
Cells in alginate beads were fixed with 2% paraformaldehyde (PFA) (Electron Microscopy Sciences) for 15 minutes at room temperature, then washed with DPBS+/+ for three times, 20 minutes each. Blocking was done with 3% bovine serum albumin (Sigma-Aldrich) /5% goat serum (Sigma-Aldrich) for overnight at 4°C. EdU detection was done using Click-iT™ EdU Cell Proliferation Kit for Imaging, Alexa Fluor™ 488 dye (Invitrogen, C10337). Cells were incubated with primary antibodies (Anti-NCAM1/CD56 antibody [EP2567Y], Abcam, #ab75813 and BD Pharmingen™ Purified Mouse Anti-Human CD45, BD Biosciences, #555480) for overnight at 4°C and Alexa Fluor conjugated secondary antibodies (F(ab')2-Goat anti-Mouse IgG (H+L) Cross-Adsorbed Secondary Antibody, Alexa Fluor™ 594 and F(ab')2-Goat anti-Rabbit IgG (H+L) Cross-Adsorbed Secondary Antibody, Alexa Fluor™ 488 - A-11070, both from Invitrogen) for overnight at 4°C protected from light. Washing was done after each incubation, with DPBS+/+ for three times, 20 minutes each.
Bead analysis
Beads were analysed using a COPAS PLUS (Union Biometrica) large particle flow sorter equipped with 488 nm and 561 solid state lasers and Green PMT 514/23 nm, Yellow PMT 585/20 nm, Red PMT 615/45 nm optical emission filters. The instrument was calibrated using a reference sample of beads. Sorting gates for size (TOF), optical density (EXT) and fluorescence parameters for each experiment were set using representative samples of beads that were labelled with secondary antibody only.
Hit detection and isolation.
Hit beads with high fluorescence intensities for EdU-Click+/CD45+ (Exit 1) or CD56+/CD45+ (Exit 2) cells were identified and sorted into individual wells of 96-well plate using COPAS (Union Biometrica).
Tag analysis. Individually isolated beads were dissolved to release tags by overnight incubation in Trypsin/EDTA at 37°C and then resuspended in 0.1% Tween in PBS. Tags used in the experiment comprised 25 unique populations of fluorescent microspheres distinguished by size and fluorescent intensity. Tags were analysed using BD FACSCanto^{TM} II flow cytometer (BD Biosciences). Before tag analysis, a reference tag set was used to establish side and forward scattering gates and to calibrate fluorescence intensity of each tag set. Tag identification was performed using Ariadne^{TM} bioinformatics software (proprietary to Plasticell Ltd.).
Ariadne^{TM} and component analysis. Probability values for the occurrence of given events by chance were obtained from computer simulation experiments. A Mersenne Twister random number generator was used to output uniformly distributed 32-bit integers, which were scaled to cover 10,000, 1,000, or 100 possible pathways, when simulating common cell culture media on four of four splits, three of four splits or two of four splits. Simulation begins by setting tally counters associated with each pathway to zero. Event probabilities were computed by repeating the process 100 million times and dividing the number of positive results by the total number of simulations, resulting in probability values accurate to eight decimal places.

2. Flow cytometry reagents

All reagents used for the staining of iNK cells were as following:

**Item**	**Supplier**	**Catalog number**
Alexa Fluor® 488 anti-human CD3 Antibody	Biolegend	300415
Alexa Fluor® 488 anti-human CD14 Antibody	Biolegend	301811
Alexa Fluor® 488 anti-human CD19 Antibody	Biolegend	302219

APC anti-human CD335 (NKp46) Antibody	Biolegend	331918
APC/Cyanine7 anti-human Perforin Antibody	Biolegend	308128
BD Horizon™ BV421 Mouse Anti-Human CD43	BD Biosciences	562916
BD Horizon™ BV421 Mouse Anti-Human CD56	BD Biosciences	562751
BD Pharmingen™ APC Mouse Anti-Human CD34	BD Biosciences	555824
BD Pharmingen™ APC Mouse Anti-Human CD45	BD Biosciences	555485
BD Pharmingen™ FITC Mouse Anti-Human CD3	BD Biosciences	555332
BD Pharmingen™ FITC Mouse Anti-Human CD31	BD Biosciences	555445
BD Pharmingen™ FITC Mouse Anti-Human CD107a	BD Biosciences	555800
BD Pharmingen™ PE Mouse Anti-Human CD45	BD Biosciences	555483
Brilliant Violet 421™ anti-human IFN-γ Antibody	Biolegend	502532
Brilliant Violet 605™ anti-human CD57 Recombinant Antibody	Biolegend	393304
Brilliant Violet 650™ anti-human CD16 Antibody	Biolegend	302042
Brilliant Violet 711™ anti-human CD226 (DNAM-1) Antibody	Biolegend	338334
Brilliant Violet 750™ anti-human TNF-α Antibody	Biolegend	502960
Brilliant Violet 785™ anti-human CD69 Antibody	Biolegend	310932
CD56 (NCAM) Monoclonal Antibody (TULY56), eFluor™ 506, eBioscience™	Invitrogen	69-0566-42
CD159a (NKG2A) Antibody, anti-human, Vio® Bright R720, REAfinity™	Miltenyi	130-128-577
eBioscience™ 7-AAD Viability Staining Solution	Invitrogen	00-6993-50
PE anti-human TIGIT (VSTM3) Antibody	Biolegend	372704
PerCP/Cyanine5.5 anti-RANTES (CCL5) Antibody	Biolegend	515508
PE/Cyanine5 anti-human CD314 (NKG2D) Antibody	Biolegend	320844
PE/Fire™ 640 anti-human CD366 (Tim-3) Antibody	Biolegend	345062
PE/Fire™ 810 anti-human CD336 (NKp44) Antibody	Biolegend	325127
Spark UV™ 387 anti-human CD45 Antibody	Biolegend	304086
Zombie UV™ Fixable Viability Kit	Biolegend	423107

3. Determination of purity of iNK cell product following manufacturing run

iPSC-derived hematopoietic progenitor cells (HPC) (Day 15) and iNK cells (Days 26 and 35) were collected, centrifuged at 500g for 2 minutes and resuspended at 10^6 viable cells/mL. On each time point, 100 µL of the cell suspension was collected and resuspended in 50 µL of pre-prepared surface antibody cocktails (Day 15 - CD34, APC Mouse Anti-Human, Clone 581; CD31, FITC Mouse Anti-Human, Clone WM59; CD43, BV421 Mouse Anti-Human, Clone 1G10; CD45, PE Mouse Anti-Human, Clone HI30. Days 26 and 35 - CD45, APC Mouse Anti-Human, Clone HI30; CD56, BV421 Mouse Anti-Human, Clone NCAM16.2; CD3,

FITC Mouse Anti-Human, Clone UCHT1) and their corresponding Fluorescence Minus One antibody cocktails to be used as negative controls, and incubated for 20 minutes at 4 °C. Post incubation, 150 µL of FACS buffer was added and centrifuged at 500g for 2 minutes at room temperature. For viability staining, cell samples were resuspended in a viability dye 7-AAD working solution (1:20 dilution of viability dye 7-AAD in DPBS-/-) and incubated for 5 minutes at room temperature, protected from light. Each sample was diluted with 100 µL FACS buffer. 10,000 cells per samples were acquired to analyse stage-specific QC parameters on single cells after doublet and live/dead cell discrimination. Data were acquired using a MACSQuant flow cytometer (Miltenyi Biotec) and analysed using the FlowJo v10 software.

Supplementary Table 1: CombiCult® Matrix media compositions

<table>
<tr><th rowspan="2">Ste p</th><th rowspan="2">Conditio n</th><th colspan="2">Media Composition</th></tr>
<tr><th>Basal Media + Common supplements</th><th>Unique supplements</th></tr>
<tr><td rowspan="8">1</td><td>1</td><td rowspan="8">APEL2
ROCKi (10uM)
B-mercaptoethanol (25uM)
SCF (40ng/ml)
L-AA (200uM)
P/S (1%)
BMP4 (20ng/ml)</td><td>Rapamycin (1uM)
CHIR99021 (3uM)
VEGF (20ng/ml)</td></tr>
<tr><td>2</td><td>CHIR99021 (Day 2-3) (3uM)
VEGF (20ng/ml)</td></tr>
<tr><td>3</td><td>Activin A (25ng/ml)
CHIR99021 (3uM)</td></tr>
<tr><td>4</td><td>Activin A (15ng/ml)
LiCl (2mM)
bFGF (5ng/ml)</td></tr>
<tr><td>5</td><td>CHIR99021 (3uM)
DEAB (10uM)
VEGF (20ng/ml)</td></tr>
<tr><td>6</td><td>CHIR99021 (3uM)
bFGF (5ng/ml)
VEGF (20ng/ml)</td></tr>
<tr><td>7</td><td>bFGF (5ng/ml)
VEGF (20ng/ml)</td></tr>
<tr><td>8</td><td>Pifithrin (2uM)
DM (10uM)
CHIR99021 (3uM)</td></tr>
<tr><td></td><td></td><td></td><td></td></tr>
<tr><td rowspan="9">2</td><td>1</td><td rowspan="9">APEL2
B-mercaptoethanol (25uM)
SCF (40ng/ml)
L-AA (200uM)
P/S (1%)
BMP4 (20ng/ml)
VEGF (20ng/ml)</td><td>SB431542 (10uM)
bFGF (5ng/ml)</td></tr>
<tr><td>2</td><td>XMU-MP-1 (1uM)
bFGF (5ng/ml)</td></tr>
<tr><td>3</td><td>Jagged1 Fc (500ng/ml)
Estradiol (10nM)</td></tr>
<tr><td>4</td><td>DLL4 Fc (1ug/ml)
IGF-1 (100ng/ml)
bFGF (5ng/ml)</td></tr>
<tr><td>5</td><td>Retinoic Acid (1nM)
bFGF (5ng/ml)
Valproic Acid (1.74uM)
IBMX (50uM)</td></tr>
<tr><td>6</td><td>IGF-1 (100ng/ml)
Estradiol (10nM)
DLL4 Fc (1ug/ml)</td></tr>
<tr><td>7</td><td>IL-3 (5ng/ml)
IL-6 (10ng/ml)
TPO (10ng/ml)</td></tr>
<tr><td>8</td><td>Nicotinamide (2.5mM)
Tryptophan (0.1uM)</td></tr>
<tr><td>9</td><td>ML 228 (0.5uM)</td></tr>
</table>

			IL-3 (1ng/ml)<br>IL-6 (10ng/ml)<br>TPO (10ng/ml)<br>FLT3L (10ng/ml)<br>Pifithrin (2uM)
3	1	APEL2<br>B-mercaptoethanol (25uM)<br>SCF (40ng/ml)<br>L-AA (200uM)<br>P/S (1%)<br>IL-3 (5ng/ml)<br>IL-7 (20ng/ml)<br>IL-15 (10ng/ml)<br>BMP4 (20ng/ml)<br>VEGF (20ng/ml)	DLL4 Fc (1ug/ml)<br>IGF-1 (100ng/ml)<br>bFGF (5ng/ml)
	2		bFGF (5ng/ml)<br>SB431542 (10uM)
	3		Estradiol (10nM)<br>IGF-1 (100ng/ml)<br>FLT3L (10ng/ml)<br>SDF1 (200ng/ml)
	4		Jagged 1 Fc (500ng/ml) + anti-IgG Ab<br>SR1 (1uM)
	5		SR1 (1uM)<br>IL-6 (10ng/ml)<br>TPO (10ng/ml)<br>Sushi (1:1 ratio with IL-15)
	6		IL-6 (10ng/ml)<br>TPO (10ng/ml)<br>FLT3L (100ng/ml)
	7		Nicotinamide (2.5mM)<br>Tryptophan (0.1uM)<br>DLL4 Fc (1ug/ml) + anti-IgG Ab
	8		IL-6 (10ng/ml)<br>TPO (10ng/ml)
4	1	DMEM<br>F12<br>B-mercaptoethanol<br>L-Glutamine<br>Sodium Selenite<br>Ethanolamine<br>L-AA (200uM)<br>SCF (40ng/ml)<br>IL-7 (20ng/ml)<br>IL-15 (10ng/ml)<br>FLT3L 10ng/ml)	DLL4 Fc (1ug/ml)<br>Hydrocortisone(1uM)<br>Sushi<br>B15
	2		IGF-1 (100ng/ml)<br>TPO (10ng/ml)<br>IL-21 (10ng/ml)<br>SIS3 (0.125ng/ml) (add 1week later)<br>B15
	3		Nicotinamide (2.5mM)<br>DLL4 Fc (1ug/ml) + anti-IgG Ab<br>B15
	4		Hydrocortisone (1uM)<br>IL-2 (10ng/ml)<br>Serum
	5		Serum+ Sushi (1:1 ratio with IL-15)
	6		Serum
	7		B15

Supplementary Table 2: Media formulations for successful iNK production protocols

	**Media 1, Day 0**<br>**(Half media change – Day 2)**
**Protocol 1**	APEL2<br>P/S<br>B-mercaptoethanol (25uM)<br>L-AA (200uM)<br>ROCKi (10uM)<br>BMP4 (20ng/ml)<br>SCF (40ng/ml)<br>CHIR99021 (3uM)<br>Activin A (25ng/ml)
**Protocol 2**	APEL2<br>P/S<br>B-mercaptoethanol (25uM)<br>L-AA (200uM)<br>ROCKi (10uM)<br>BMP4 (20ng/ml)<br>SCF (40ng/ml)<br>CHIR99021 (3uM)<br>Activin A (25ng/ml)
**Protocol 3**	APEL2<br>P/S<br>B-mercaptoethanol (25uM)<br>L-AA (200uM)<br>ROCKi (10uM)<br>BMP4 (20ng/ml)<br>SCF (40ng/ml)<br>Activin A (15ng/ml)<br>LiCl (2mM)<br>bFGF (10ng/ml)
**Protocol 4**	APEL2<br>P/S<br>B-mercaptoethanol (25uM)<br>L-AA (200uM)<br>ROCKi (10uM)<br>BMP4 (20ng/ml)<br>SCF (40ng/ml)<br>CHIR99021 (3uM)<br>Activin A (25ng/ml)
**Protocol 5**	APEL2<br>P/S<br>B-mercaptoethanol (25uM)<br>L-AA (200uM)

	ROCKi (10uM)<br>BMP4 (20ng/ml)<br>SCF (40ng/ml)<br>CHIR99021 (3uM)<br>Activin A (25ng/ml)
**Protocol 6**	APEL2<br>P/S<br>B-mercaptoethanol (25uM)<br>L-AA (200uM)<br>ROCKi (10uM)<br>BMP4 (20ng/ml)<br>SCF (40ng/ml)<br>Activin A (15ng/ml)<br>LiCl (2mM)<br>bFGF (10ng/ml)
**Protocol 7**	APEL2<br>P/S<br>B-mercaptoethanol (25uM)<br>L-AA (200uM)<br>ROCKi (10uM)<br>BMP4 (20ng/ml)<br>SCF (40ng/ml)<br>CHIR99021 (3uM)<br>VEGF (20ng/ml)<br>bFGF (10ng/ml)

	**Media2, Day 3**
**Protocol 1**	APEL2<br>P/S<br>B-mercaptoethanol (25uM)<br>L-AA (200uM)<br>BMP4 (20ng/ml)<br>VEGF (20ng/ml)<br>SCF (40ng/ml)<br>SB431542 (10uM)<br>bFGF (10ng/ml)
**Protocol 2**	APEL2<br>P/S<br>B-mercaptoethanol (25uM)<br>L-AA (200uM)<br>BMP4 (20ng/ml)<br>VEGF (20ng/ml)<br>SCF (40ng/ml)

	SB431542 (10uM)<br>bFGF (10ng/ml)
**Protocol 3**	APEL2<br>P/S<br>B-mercaptoethanol (25uM)<br>L-AA (200uM)<br>BMP4 (20ng/ml)<br>VEGF (20ng/ml)<br>SCF (40ng/ml)<br>DLL4 Fc (500ng/ml)<br>IGF-1 (100ng/ml)<br>bFGF (10ng/ml)
**Protocol 4**	APEL2<br>P/S<br>B-mercaptoethanol (25uM)<br>L-AA (200uM)<br>BMP4 (20ng/ml)<br>VEGF (20ng/ml)<br>SCF (40ng/ml)<br>Nicotinamide (2.5mM)<br>Tryptophan (0.1uM)
**Protocol 5**	APEL2<br>P/S<br>B-mercaptoethanol (25uM)<br>L-AA (200uM)<br>BMP4 (20ng/ml)<br>VEGF (20ng/ml)<br>SCF (40ng/ml)<br>SB431542 (10uM)<br>bFGF (10ng/ml)
**Protocol 6**	APEL2<br>P/S<br>B-mercaptoethanol (25uM)<br>L-AA (200uM)<br>BMP4 (20ng/ml)<br>VEGF (20ng/ml)<br>SCF (40ng/ml)<br>IL-3 (5ng/ml)<br>IL-6 (10ng/ml)<br>TPO (10ng/ml)
**Protocol 7**	APEL2<br>P/S<br>B-mercaptoethanol (25uM)<br>L-AA (200uM)<br>BMP4 (10ng/ml)

	VEGF (20ng/ml)<br>SCF (40ng/ml)<br>IL-3 (5ng/ml)<br>IL-6 (10ng/ml)<br>TPO (10ng/ml)

	**Media 3, Day 6**<br>**(Half media change - Days 8,10)**
**Protocol 1**	APEL2<br>P/S<br>B-mercaptoethanol (25uM)<br>L-AA (200uM)<br>BMP4 (20ng/ml)<br>VEGF (20ng/ml)<br>SCF (40ng/ml)<br>IL-3 (5ng/ml)<br>IL-7 (20ng/ml)<br>IL-15 (10ng/ml)<br>SB431542 (10uM)<br>bFGF (10ng/ml)
**Protocol 2**	APEL2<br>P/S<br>B-mercaptoethanol (25uM)<br>L-AA (200uM)<br>BMP4 (20ng/ml)<br>VEGF (20ng/ml)<br>SCF (40ng/ml)<br>IL-3 (5ng/ml)<br>IL-7 (20ng/ml)<br>IL-15 (10ng/ml)<br>SB431542 (10uM)<br>bFGF (10ng/ml)
**Protocol 3**	APEL2<br>P/S<br>B-mercaptoethanol (25uM)<br>L-AA (200uM)<br>BMP4 (20ng/ml)<br>VEGF (20ng/ml)<br>SCF (40ng/ml)<br>IL-3 (5ng/ml)<br>IL-7 (20ng/ml)<br>IL-15 (10ng/ml)<br>Nicotinamide (2.5mM)<br>Tryptophan (0.1uM)<br>DLL4 Fc (500ng/ml)<br>Anti-IgG Ab (3.5ug/ml)

**Protocol 4**	APEL2<br>P/S<br>B-mercaptoethanol (25uM)<br>L-AA (200uM)<br>BMP4 (20ng/ml)<br>VEGF (20ng/ml)<br>SCF (40ng/ml)<br>IL-3 (5ng/ml)<br>IL-7 (20ng/ml)<br>IL-15 (10ng/ml)<br>SB431542 (10uM)<br>bFGF (10ng/ml)
**Protocol 5**	APEL2<br>P/S<br>B-mercaptoethanol (25uM)<br>L-AA (200uM)<br>BMP4 (20ng/ml)<br>VEGF (20ng/ml)<br>SCF (40ng/ml)<br>IL-3 (5ng/ml)<br>IL-7 (20ng/ml)<br>IL-15 (10ng/ml)<br>IL-6 (10ng/ml)<br>TPO (10ng/ml)<br>SR1 (1uM)<br>Sushi (10ng/ml)
**Protocol 6**	APEL2<br>P/S<br>B-mercaptoethanol (25uM)<br>L-AA (200uM)<br>BMP4 (20ng/ml)<br>VEGF (20ng/ml)<br>SCF (40ng/ml)<br>IL-3 (5ng/ml)<br>IL-7 (20ng/ml)<br>IL-15 (10ng/ml)<br>SB431542 (10uM)<br>bFGF (10ng/ml)
**Protocol 7**	APEL2<br>P/S<br>B-mercaptoethanol (25uM)<br>L-AA (200uM)<br>BMP4 (10ng/ml)<br>VEGF (20ng/ml)<br>SCF (40ng/ml)<br>IL-3 (5ng/ml)<br>IL-7 (20ng/ml)

	IL-15 (10ng/ml)<br>IL-6 (10ng/ml)<br>TPO (10ng/ml)

	**Medium 4, Day 12**<br>**(Half media change - every 3 days)**
**Protocol 1**	DMEM<br>F12<br>P/S<br>Serum (15%)<br>L-glutamine (2mM)<br>B-mercaptoethanol (25uM)<br>Sodium selenite (5ng/ml)<br>Ethanolamine (50uM)<br>L-AA (20ug/ml) (add fresh)<br>FLT3L (10ng/ml)<br>SCF (20ng/ml)<br>IL-7 (20ng/ml)<br>IL-15 (10ng/ml)
**Protocol 2**	DMEM<br>F12<br>P/S<br>L-glutamine (2mM)<br>B-mercaptoethanol (25uM)<br>Sodium selenite (5ng/ml)<br>Ethanolamine (50uM)<br>L-AA (20ug/ml) (add fresh)<br>FLT3L (10ng/ml)<br>SCF (20ng/ml)<br>IL-7 (20ng/ml)<br>IL-15 (10ng/ml)<br>B15
**Protocol 3**	DMEM<br>F12<br>P/S<br>L-glutamine (2mM)<br>B-mercaptoethanol (25uM)<br>Sodium selenite (5ng/ml)

	Ethanolamine (50uM)<br>L-AA (20ug/ml)<br>FLT3L (10ng/ml)<br>SCF (20ng/ml)<br>IL-7 (20ng/ml)<br>IL-15 (10ng/ml)<br>DLL4 Fc (500ng/ml)<br>Hydrocortisone (1uM)<br>Sushi (10ng/ml)<br>B15
**Protocol 4**	DMEM<br>F12<br>P/S<br>L-glutamine (2mM)<br>B-mercaptoethanol (25uM)<br>Sodium selenite (5ng/ml)<br>Ethanolamine (50uM)<br>L-AA (20ug/ml)<br>FLT3L (10ng/ml)<br>SCF (20ng/ml)<br>IL-7 (20ng/ml)<br>IL-15 (10ng/ml)<br>Nicotinamide (2.5mM)<br>DLL4 Fc (500ng/ml)<br>Anti-IgG Ab (3.5ug/ml)<br>B15
**Protocol 5**	DMEM<br>F12<br>P/S<br>L-glutamine (2mM)<br>B-mercaptoethanol (25uM)<br>Sodium selenite (5ng/ml)<br>Ethanolamine (50uM)<br>L-AA (20ug/ml)<br>FLT3L (10ng/ml)<br>SCF (20ng/ml)<br>IL-7 (20ng/ml)<br>IL-15 (10ng/ml)<br>Nicotinamide (2.5mM)<br>DLL4 Fc (500ng/ml)<br>Anti-IgG Ab (3.5ug/ml)<br>B15
**Protocol 6**	DMEM

	F12<br>P/S<br>L-glutamine (2mM)<br>B-mercaptoethanol (25uM)<br>Sodium selenite (5ng/ml)<br>Ethanolamine (50uM)<br>L-AA (20ug/ml)<br>FLT3L (10ng/ml)<br>SCF (20ng/ml)<br>IL-7 (20ng/ml)<br>IL-15 (10ng/ml)<br>DLL4 Fc (500ng/ml)<br>Hydrocortisone (1uM)<br>Sushi (10ng/ml)<br>B15
**Protocol 7**	DMEM<br>F12<br>P/S<br>Serum (15%)<br>L-glutamine (2mM)<br>B-mercaptoethanol (25uM)<br>Sodium selenite (5ng/ml)<br>Ethanolamine (50uM)<br>L-AA (20ug/ml) (add fresh)<br>FLT3L (10ng/ml)<br>SCF (20ng/ml)<br>IL-7 (20ng/ml)<br>IL-15 (10ng/ml)

**B15**	PVA (0.5%)<br>Cellastim-S (0.25%)<br>rAlbumin (2.5%)<br>ITS-X (1X)<br>NEAA (1X)<br>SyntheChol NS0 Supplement (1X)<br>Progesterone (3.15ng/ml)<br>Corticosterone (10ng/ml)<br>Linoleic acid (500ng/ml)<br>Linolenic acid (500ng/ml)<br>Retinyl acetate (50ng/ml)<br>L-carnitine (1ug/ml)<br>Vitamin B12 (340ng/ml)
